# Prevalence and spectrum of *AKT1*, *PIK3CA*, *PTEN* and *TP53* somatic mutations in Chinese breast cancer patients

**DOI:** 10.1371/journal.pone.0203495

**Published:** 2018-09-13

**Authors:** Guoli Li, Xinwu Guo, Ming Chen, Lili Tang, Hui Jiang, Julia X. Day, Yueliang Xie, Limin Peng, Xunxun Xu, Jinliang Li, Shouman Wang, Zhi Xiao, Lizhong Dai, Jun Wang

**Affiliations:** 1 School of Life Sciences, Central South University, Changsha, Hunan, China; 2 Sanway Gene Technology Inc., Changsha, Hunan, China; 3 Department of Breast Surgery, Xiangya Hospital, Central South University, Changsha, Hunan, China; 4 Research Center for Technologies in Nucleic Acid-Based Diagnostics, Changsha, Hunan, China; 5 Research Center for Technologies in Nucleic Acid-Based Diagnostics and Therapeutics, Changsha, Hunan, China; CNR, ITALY

## Abstract

Breast cancer, one of the most frequently occurring cancers worldwide, is the leading cause of cancer-related death among women. *AKT1*, *PIK3CA*, *PTEN* and *TP53* mutations were common observed in breast cancer representing potential clinical biomarkers for cancer classification and treatment. A comprehensive knowledge of *AKT1*, *PIK3CA*, *PTEN* and *TP53* mutations in breast cancer was still insufficient in Chinese population. In this study, the complete coding regions and exon-intron boundaries of *AKT1*, *PIK3CA*, *PTEN* and *TP53* genes were sequenced in paired breast tumor and normal tissues from 313 Chinese breast cancer patients using microfluidic PCR-based target enrichment and next-generation sequencing technology. Total 120 somatic mutations were identified in 190 of the 313 patients (60.7%), with the mutation frequency of *AKT1* as 3.2%, *PIK3CA* as 36.4%, *PTEN* as 4.8%, and *TP53* as 33.9%. Among these mutations, 1 in *PIK3CA* (p.I69N), 3 in *PTEN* (p.K62X, c.635-12_636delTTAACCATGCAGAT and p.N340IfsTer4) and 5 in *TP53* (p.Q136AfsTer5, p.K139_P142del, p.Y234dup, p.V274LfsTer31 and p.N310TfsTer35) were novel. Notably, *PIK3CA* somatic mutations were significantly associated with ER-positive or PR-positive tumors. *TP53* somatic mutations were significantly associated with ER-negative, PR-negative, HER2-positive, *BRCA1* mutation, Ki67 high expression and basal-like tumors. Our findings provided a comprehensive mutation profiling of *AKT1*, *PIK3CA*, *PTEN* and *TP53* genes in Chinese breast cancer patients, which have potential implications in clinical management.

## Introduction

Breast cancer is the most common cancer types and the leading cause of cancer mortality in females in the world [1]. It was estimated that approximately 278,800 new breast cancer cases with 64,600 deaths occurred in China in 2013 [2]. It is well known that cancer progression is driven by mutations in cancer genome [3]. Somatic mutations in *AKT1*, *PIK3CA PTEN* and *TP53* genes were found at high frequency in breast cancer, with *PIK3CA* as 26.4%, *TP53* as 24.7%, *PTEN* as 3.8% and *AKT1* as 2.8% in the Catalogue of Somatic Mutations in Cancer (COSMIC) database [4]. Recent large genomic landscape studies have showed that *TP53* and *PIK3CA* were the two most frequently mutated driver genes in primary breast cancer and the mutation spectrum of these four genes displayed subgroup specificity with great clinical significance in cancer classification and treatment [5, 6]. However, the spectrum of these four gene mutations in breast cancer is still largely unknown in Chinese population. Thus a comprehensive understanding of the prevalence and clinical characteristics of *AKT1*, *PIK3CA*, *PTEN* and *TP53* gene mutations in Chinese breast cancer patients is urgently needed.

With the advance of next-generation sequencing (NGS) technologies, mutation analysis has become effective and feasible for routine clinical application in breast cancer [7]. In this study, paired tumor and normal tissues from a cohort of 313 Chinese breast cancer patients were screened for *ATK1*, *PIK3CA*, *PTEN* and *TP53* mutations using microfluidic PCR-based target enrichment and NGS technology. Furthermore, clinicopathological characteristics of breast cancer associated with the mutations of these four genes were analyzed in parallel.

## Material and methods

### Patients and tissue samples

Fresh tumor and paired adjacent normal tissues (located at least 2 cm away from the site of tumor tissue) from 313 primary breast cancer patients were collected at Xiangya Hospital, Central South University from year 2013 to 2015. The clinicopathological characteristics of the 313 patients were summarized in Table 1. All breast specimens were reviewed by experienced pathologists. The breast cancer molecular subtypes were characterized based on the guideline of St Gallen International Expert Consensus (2013) [8]. All of the 313 patients have been tested for *BRCA1* and *BRCA2* mutations by NGS and validated using Sanger sequencing in our previous study [9]. All the patients in this study were females of Chinese Han population without selection for family history or onset age. We declared that the experiments performed in this study comply with the current laws of the People's Republic of China. This study was approved by the Ethics Committee of Central South University, Changsha, China, and all participants had given written informed consent.

**Table 1:** Clinicopathological characteristics of 313 breast cancer patients.

Characteristics		Number of patients, n (%)
**Age at diagnosis**	<35	10 (3.2%)
	<40	27 (8.6%)
	<50	145 (46.3%)
	≥50	168 (53.7%)
	Mean	51.24 (21–84)
**Lymph node metastasis**	Positive(+)	137 (43.8%)
	Negative(-)	170 (54.3%)
	unknown	6 (1.9%)
**ER status**	Positive(+)	222 (70.9%)
	Negative(-)	90 (28.8%)
	unknown	1 (0.3%)
**PR status**	Positive(+)	163 (52.1%)
	Negative(-)	148 (47.3%)
	unknown	2 (0.6%)
**HER2 status**	Positive(+)	89 (28.4%)
	Negative(-)	177 (56.6%)
	unknown	47 (15.0%)
**p53 mutation** **(IHC)**	Positive(+)	239 (76.4%)
	Negative(-)	64 (20.4%)
	unknown	10 (3.2%)
**Ki67 over-expression**	<14%	134 (42.8%)
	≥14%	177 (56.6%)
	unknown	2 (0.6%)
**Tumor grade**	I	11 (3.5%)
	II	247 (78.9%)
	III or IV	19 (6.1%)
	Unknown	36 (11.5%)
**Tumor type**	IDC	221 (70.6%)
	ILC	5 (1.6%)
	Mucinous	7 (2.2%)
	Others	80 (25.6%)
**Molecular subtype**	Luminal A	86 (27.5%)
	Luminal B	128 (40.9%)
	Basal-like	40 (12.8%)
	HER2-enriched	37 (11.8%)
	unknown	22 (7.0%)

ER, estrogen receptor; PR, progesterone receptor; HER2, human epidermal growth factor receptor 2; IDC, invasive ductal carcinoma; ILC, invasive lobular carcinoma; IHC, immunohistochemistry.

### Library preparation and NGS

Genomic DNA of all samples were extracted using the TIANamp Genomic DNA Kit (TianGen Biotech, Beijing, China), and quantified using a Nanodrop 2000 spectrophotometer (Thermo Scientific, Wilmington, DE, USA). Totally, 60 pairs of primers were designed to amplify the complete coding regions and exon–intron boundaries of target genes, and the primer sequences were displayed in S1 Table. The primers were designed using the on-line design tool, Primer3 (http://bioinfo.ut.ee/primer3/), by following the User Guide of Access Array^TM^ System for Illumina Sequencing Systems (Fluidigm, South San Franciso, CA, USA). All primers were validated by single-plex PCR with assessment of PCR products for expected size on agarose gel. The high-throughput target enrichment was performed on the Fluidigm Access Array (Fluidigm, South San Franciso, CA, USA) according to established workflows [9–11]. Then the target gene libraries were sequenced on an Illumina MiSeq sequencer (SanDiego, CA, USA) using MiSeq Reagent Kit v2 (500 cycles).

### Sequencing data analysis and variant annotation

The raw sequencing data were base-called and demultiplexed using MiSeq Reporter v.1.8.1 (Illumina, SanDiego, CA, USA) with default parameters and FASTQ files were generated for downstream data analysis. The adapter sequences and low quality reads were trimmed away from the raw reads using Trimmomatic v.0.32 [12]. Cleaned reads were aligned to the UCSC human reference genome hg19 using the Burrows-Wheeler Alignment tool (BWA) v.0.7.10 [13]. After alignment, the SAMtools (v.1.1) [14] software was applied to convert the alignment files to a sorted, indexed binary alignment map (BAM) format. Base recalibration and realignment around indels was done with the GATK v3.1.1 [15]. Germline genotypes were called by the GATK UnifiedGenotyper (with paired tumor and adjacent normal tissues sample), and somatic mutations were called by MuTect (v.1.1.4) [16] under the High-Confidence mode with default parameter settings. Both tumor and matched normal tissue samples from the same patient were sequenced together in a NGS run. The variants present only in tumor tissue samples were thus classified as somatic mutations. And variants present in both tumor and paired normal tissue samples were classified as germline mutations. We defined the final list of somatic mutations with the following filters: number of reads with the altered base in the tumor ≥10; frequency of the reads with the altered base in the tumor ≥ 5% except for variants that are also reported in COSMIC database; minor allele frequency <0.1% in each of the following publicly available databases: 1000 Genomes (http://www.1000genomes.org/) and Exome Aggregation Consortium (http://exac.broadinstitute.org/). Variants were annotated using ANNOVAR (February, 2016) with the annotate_variation.pl script [17]. This tool mapped variants to RefSeq genes, known variations from dbSNP 138 and annotated the predicted functional consequences of missense variants using two *silico* tools (SIFT [18] and PolyPhen-2 [19]). Additional clinical variant annotations were obtained from NCBI ClinVar (http://www.ncbi.nlm.nih.gov/clinvar), and COSMIC database (http://cancer.sanger.ac.uk/cosmic). The reference sequences for numbering were based on the NCBI GenBank Database for *AKT1* (NM_005163.2 and NP_005154.2), *PIK3CA* (NM_006218.2 and NP_006209.2), *PTEN* (NM_000314.4 and NP_000305.3) and *TP53* (NM_000546.5 and NP_000537.3). Novel mutations were defined as variants that have neither been previously recorded in dbSNP (http://www.ncbi.nlm.nih.gov/SNP), ClinVar (http://www.ncbi.nlm.nih.gov/clinvar/), 1000 Genomes (http://www.1000genomes.org/), Exome Aggregation Consortium (http://exac.broadinstitute.org/) or COSMIC (http://cancer.sanger.ac.uk/cosmic), nor reported in literatures. In this study, all variants were classified according to the American College of Medical Genetics and Genomics recommendations [20]. Variants resulted in non-functional or truncating-proteins were classified as pathogenic mutations (including stop-gain mutations, frameshift mutations and splice site mutations). In addition, we also considered variants as pathogenic mutations if they were annotated as “pathogenic” in NCBI ClinVar. The annotation and classification of the protein domains of these 4 genes was based on the NCBI’s Conserved Domain Database (CDD) [21].

### Statistical analysis

Continuous data were summarized using mean and standard deviation. The difference of age among patients with different gene mutation status was determined by the Wilcoxon Rank Sum test. And χ^2^ test was used to compare categorical variables between groups across clinicopathological characteristics. Alternatively, Fisher’s exact test was used when χ^2^ test was violated. The obtained *P* values were considered statistically significant if the *P* value is < 0.05. The Holm’s procedure was used to adjust *P* values for multiple testing [22]. All the computations were performed using the R software (version 3.1.0, http://www.cran.r-project.org).

## Results

### Detection of mutations by NGS

Microfluidic PCR-based target enrichment and NGS were performed to sequence the entire coding regions and exon-intron boundaries of *AKT1*, *PIK3CA*, *PTEN* and *TP53* genes in the cohort of 313 Chinese breast cancer patients. Total 120 somatic mutations were detected in 190 patients (190/313, 60.7%) (Table 2 and S2 Table). The somatic mutation frequency of *AKT1*, *PIK3CA*, *PTEN* and *TP53* in this cohort was 3.2% (10/313), 36.4% (114/313), 4.8% (15/313) and 33.9% (106/313), respectively. Similarly, the somatic mutation frequency of these genes reported by TCGA [23] was 2.4%, 35.5%, 3.2% and 35.3%, respectively (Table 2). Notably, one synonymous variant (p.T125T in *TP53*) was included in this study, because it can lead to alternative splicing as previously reported [24]. In addition, 6 germline mutations were also found (1 in *PIK3CA*, 2 in *PTEN* and 3 in *TP53*) in 6 of the 313 patients (S3 Table). Among these 126 mutations, 53 were considered as pathogenic (42.9%), including 52 somatic mutations and 2 germline mutations (Table 3). All the somatic mutations detected in this study were confirmed in two different NGS runs. In addition, all germline mutations and the somatic mutations with allele fraction ≧20% in tumor tissues were confirmed using Sanger sequencing (S1 and S2 Figs).

**Table 2:** Frequencies of somatic mutations in this study compared with TCGA data.

Mutation pattern	In this study of 313 patients		In TCGA data (n = 507)	
	# patients	Percentage	# patients	Percentage
**Mutation in gene**				
*AKT1*	10	3.2%	23	2.4%
*PIK3CA*	114	36.4%	179	35.3%
*PTEN*	15	4.8%	16	3.2%
*TP53*	106	33.9%	179	35.3%
**Mutation in single gene**				
*AKT1*	6	1.9%	9	1.8%
*PIK3CA*	66	21.1%	130	25.6%
*PTEN*	6	1.9%	6	1.2%
*TP53*	59	18.8%	127	25.0%
**Co-mutation in two genes**				
*AKT1*+*PIK3CA*	2	0.6%	1	0.2%
*AKT1*+*PTEN*	0	0.0%	0	0.0%
*AKT1*+*TP53*	2	0.6%	2	0.4%
*PIK3CA*+*PTEN*	4	1.3%	4	0.8%
*PIK3CA*+*TP53*	40	12.8%	44	8.7%
*PTEN*+*TP53*	3	1.0%	6	1.2%
**Co-mutation in three genes**				
*AKT1*+*PIK3CA*+*TP53*	0	0.0%	0	0.0%
*PIK3CA*+*PTEN*+*TP53*	2	0.6%	0	0.0%
**At least one mutation in *AKT1*/*PIK3CA*/*PTEN***	131	41.9%	202	39.8%
**At least one mutation in *AKT1*/*PIK3CA*/*PTEN*/*TP53***	190	60.7%	329	64.9%

TCGA: The Cancer Genome Atlas (Nature, 2012, 490(7418): 61–70.)

**Table 3:** Pathogenic mutations of *AKT1*, *PIK3CA*, *PTEN* and *TP53* genes in the 313 breast cancer patients.

Gene	Nucleotide change ^a^	Effect on protein ^a^	Mut Type ^b^	Previously reported ^c^	#Patients	Frequency	Status
***AKT1***	c.49G>A	p.E17K	Missense	dbSNP|COSMIC|ClinVar|ExAC	9	2.88%	Somatic
***PIK3CA***	c.1256_1261delACTGTC	p.H419_C420del	Inframe del	COSMIC	1	0.32%	Somatic
	c.1258T>C	p.C420R	Missense	dbSNP|COSMIC|ClinVar	2	0.64%	Somatic
	c.1624G>A	p.E542K	Missense	dbSNP|COSMIC|ClinVar	7	2.24%	Somatic
	c.1633G>A	p.E545K	Missense	dbSNP|COSMIC|ClinVar|ExAC	26	8.31%	Somatic
	c.1634A>G	p.E545G	Missense	dbSNP|COSMIC|ClinVar	1	0.32%	Somatic
	c.3140A>G	p.H1047R	Missense	dbSNP|COSMIC|ClinVar|ExAC	52	16.61%	Somatic
	c.3140A>T	p.H1047L	Missense	dbSNP|COSMIC|ClinVar|ExAC	15	4.79%	Somatic
***PTEN***	c.45_46insT	p.Y16LfsTer28	Frameshift ins	COSMIC	1	0.32%	Somatic
	c.49C>T	p.Q17X	Nonsense	dbSNP|COSMIC|ClinVar	1	0.32%	Somatic
	c.79T>A	p.Y27N	Missense	dbSNP|COSMIC|ClinVar|ExAC	1	0.32%	Somatic
	c.184A>T	p.K62X	Nonsense	**Novel**	1	0.32%	Somatic
	c.406T>C	p.C136R	Missense	dbSNP|ClinVar	1	0.32%	**Germline**
	c.601G>T	p.E201X	Nonsense	COSMIC	1	0.32%	Somatic
	c.633C>A	p.C211X	Nonsense	dbSNP|COSMIC|ClinVar	1	0.32%	Somatic
	c.635-12_636delTTAACCATGCAGAT	-	Splicing	**Novel**	1	0.32%	Somatic
	c.697C>T	p.R233X	Nonsense	dbSNP|COSMIC|ClinVar	1	0.32%	Somatic
	c.892C>T	p.Q298X	Nonsense	dbSNP|COSMIC|ClinVar|ExAC	1	0.32%	Somatic
	c.955_958delACTT	p.T319Ter	Frameshift del	dbSNP|COSMIC|ClinVar	1	0.32%	Somatic
	c.1003C>T	p.R335X	Nonsense	dbSNP|COSMIC|ClinVar	1	0.32%	Somatic
	c.1008C>G	p.Y336X	Nonsense	COSMIC	1	0.32%	Somatic
	c.1019delA	p.N340IfsTer4	Frameshift del	**Novel**	1	0.32%	Somatic
***TP53***	c.166G>T	p.E56X	Nonsense	COSMIC	1	0.32%	Somatic
	c.281C>A	p.S94X	Nonsense	COSMIC	1	0.32%	Somatic
	c.310C>T	p.Q104X	Nonsense	COSMIC	1	0.32%	Somatic
	c.376-2delA	-	Splicing	COSMIC	1	0.32%	Somatic
	c.406_428delCAACTGGCCAAGACCTGCCCTGT	p.Q136AfsTer5	Frameshift del	**Novel**	1	0.32%	Somatic
	c.414_425delCAAGACCTGCCC	p.K139_P142del	Inframe del	**Novel**	1	0.32%	Somatic
	c.423_425delCCC	p.P142del	Inframe del	COSMIC	1	0.32%	Somatic
	c.469G>T	p.V157F	Missense	dbSNP|COSMIC|ClinVar|ExAC	1	0.32%	Somatic
	c.488A>G	p.Y163C	Missense	dbSNP|COSMIC|ClinVar	1	0.32%	Somatic
	c.524G>A	p.R175H	Missense	dbSNP|COSMIC|ClinVar|ExAC	6	1.92%	Somatic
	c.559+1G>A	-	Splicing	COSMIC	1	0.0032	**Germline**
	c.574C>T	p.Q192X	Nonsense	dbSNP|COSMIC|ClinVar	2	0.64%	Somatic
	c.584T>C	p.I195T	Missense	dbSNP|COSMIC|ClinVar|ExAC	2	0.64%	Somatic
	c.592G>T	p.E198X	Nonsense	COSMIC	1	0.32%	Somatic
	c.626_627delGA	p.R209KfsTer6	Frameshift del	COSMIC	1	0.32%	Somatic
	c.637C>T	p.R213X	Nonsense	dbSNP|COSMIC|ClinVar|ExAC	4	1.28%	Somatic
	c.652_654delGTG	p.V218del	Inframe del	COSMIC	1	0.32%	Somatic
	c.659A>G	p.Y220C	Missense	dbSNP|COSMIC|ClinVar|ExAC	1	0.32%	Somatic
	c.700_702dupTAC	p.Y234dup	Inframe ins	**Novel**	1	0.32%	Somatic
	c.770T>A	p.L257Q	Missense	dbSNP|COSMIC|ClinVar	1	0.32%	Somatic
	c.794T>C	p.L265P	Missense	dbSNP|COSMIC|ClinVar	1	0.32%	Somatic
	c.818G>A	p.R273H	Missense	dbSNP|COSMIC|ClinVar|ExAC	2	0.64%	Somatic
	c.820_821delGT	p.V274LfsTer31	Frameshift del	**Novel**	1	0.32%	Somatic
	c.826_831delGCCTGT	p.A276_C277del	Inframe del	COSMIC	1	0.32%	Somatic
	c.844C>T	p.R282W	Missense	dbSNP|COSMIC|ClinVar|ExAC	1	0.32%	Somatic
	c.880G>T	p.E294X	Nonsense	COSMIC	1	0.32%	Somatic
	c.916C>T	p.R306X	Nonsense	dbSNP|COSMIC|ClinVar	2	0.64%	Somatic
	c.920-1G>T	-	Splicing	COSMIC	1	0.32%	Somatic
	c.929delA	p.N310TfsTer35	Frameshift del	**Novel**	1	0.32%	Somatic
	c.958A>T	p.K320X	Nonsense	COSMIC	1	0.32%	Somatic
	c.1146delA	p.K382NfsTer40	Frameshift del	COSMIC	1	0.32%	Somatic

^a^ Variant positions were reported in *AKT1* according to NCBI RefSeq NM_005163.2 (mRNA) and NP_005154.2 (Protein), in *PIK3CA* according to NCBI RefSeq NM_006218.2 (mRNA) and NP_006209.2 (Protein), in *PTEN* according to NCBI RefSeq NM_000314.4 (mRNA) and NP_000305.3 (Protein) and in *TP53* according to NCBI RefSeq NM_000546.5 (mRNA) and NP_000537.3 (Protein).

^b^ Del, deletion; ins, insertion.

^c^ Novel variants were defined as variants that have neither been previously recorded in dbSNP (http://www.ncbi.nlm.nih.gov/SNP), ClinVar (http://www.ncbi.nlm.nih.gov/clinvar/), 1000 Genomes (http://www.1000genomes.org/), Exome Aggregation Consortium (http://exac.broadinstitute.org/) or COSMIC (http://cancer.sanger.ac.uk/cosmic), nor reported in the literature.

### Frequency and spectrum of *AKT1*, *PIK3CA*, *PTEN* and *TP53* mutations

**Fig 1:**
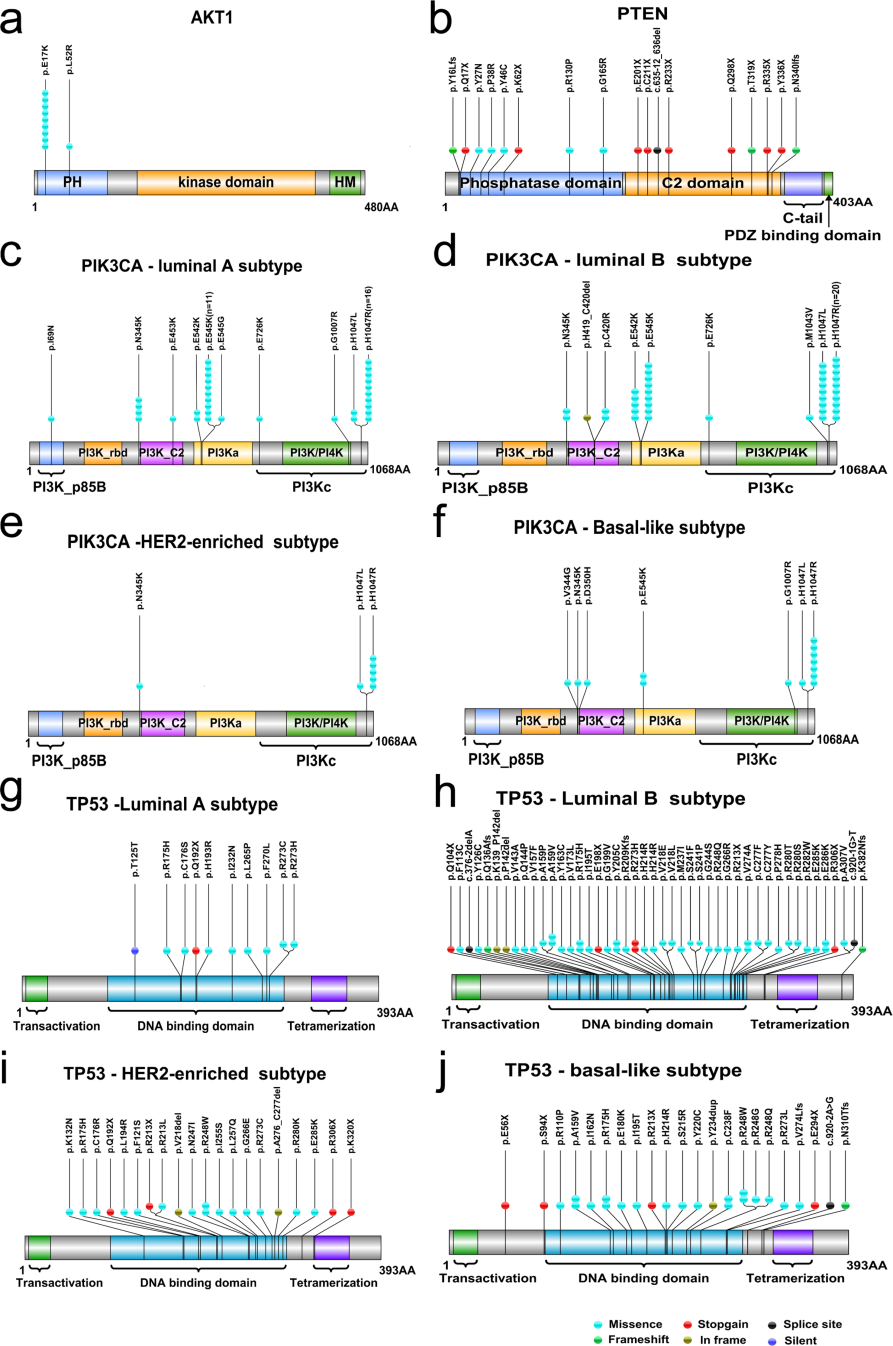
**Mutational spectrum of *AKT1* (a), *PTEN* (b), *PIK3CA* according to molecular subtypes (c-f) and *TP53* according to molecular subtypes (g-j).** Non-silent somatic mutations mapped to the protein sequence of each genes are shown. Cyan dot indicates missense mutation; Red dot indicates nonsense mutation; Black dot indicates splice site mutation; Green dot indicates frameshift mutation; Brown dot indicates in-frame mutation. The number of dots indicates the number of cases. Protein domains are shown as colored bars: PH, pleckstrin homology domain; HM, hydrophobic motif domain; C2, conserved domain 2; PI3K_p85B, p85 binding domain; PI3K_rbd, Ras-binding domain; PI3Ka, accessory domain; PI3K/PI4K, phosphatidylinositol 3-kinase and phosphatidylinositol 4-kinase domain.

In *AKT1* gene, total 2 somatic mutations were detected in 10 of 313 patients (3.2%), both of which were missense mutations located in exon 3 within pleckstrin homology (PH) domain of the AKT1 protein. The mutation p.E17K, which occurred in 9 patients (9/10, 90%), and dominated the mutation spectrum of *AKT1* (Fig 1A and S2 Table). No germline mutation was found in *AKT1*.

In *PIK3CA* gene, total 17 somatic mutations were detected in 114 of the 313 patients (36.4%), which located across 7 different exons (exon 2, 5, 8, 9, 10, 14 and 21) (S2 Table). Notably, 9 patients harbored two mutations of *PIK3CA*. Exon 10 and 21 were the two hotspot regions within PI3Ka and PI3Kc domain, mutations of which presented in 34 (34/114, 29.8%) and 70 (70/114, 61.4%) of cases, respectively (Fig 1C–1F and S2 Table). Among them, 26 patients had p.E545K mutation and 7 patients had p.E542K mutation in PI3Ka domain (Table 4). Total 52 patients had p.H1047R mutation in PI3Kc domain, and 15 patients had a different p.H1047L mutation at the same spot (Table 4). One novel somatic mutation p.I69N was found in the PI3K_p85B domain (S2 Table). In addition, one germline mutation (p.K733R) was detected in *PIK3CA*. By *in silico* analysis, it was predicted to be deleterious by SIFT and benign by PolyPhen-2 (S3 Table).

**Table 4:** Recurrent somatic mutations with the percentage >1% in the 313 breast cancer patients.

Gene	Chr	Pos	rsID	Exon	Nucleotide change	Effect on protein	# Patients	Percentage in all mutations of the gene	Percentage in the 313 patients
***AKT1***	chr14	105246551	rs121434592	exon3	c.49G>A	p.E17K	9	90.0%	2.9%
***PIK3CA***	chr3	178921553	rs121913284	exon5	c.1035T>A	p.N345K	8	6.5%	2.6%
	chr3	178936082	rs121913273	exon10	c.1624G>A	p.E542K	7	5.7%	2.2%
	chr3	178936091	rs104886003	exon10	c.1633G>A	p.E545K	26	21.1%	8.3%
	chr3	178952085	rs121913279	exon21	c.3140A>G	p.H1047R	52	42.3%	16.6%
	chr3	178952085	rs121913279	exon21	c.3140A>T	p.H1047L	15	12.2%	4.8%
***TP53***	chr17	7578454		exon5	c.476C>T	p.A159V	4	3.6%	1.3%
	chr17	7578406	rs28934578	exon5	c.524G>A	p.R175H	6	5.4%	1.9%
	chr17	7578212		exon6	c.637C>T	p.R213X	4	3.6%	1.3%
	chr17	7577539	rs121913342	exon7	c.742C>T	p.R248W	5	4.5%	1.6%
**Total**							125	55.7%	39.9%

In *PTEN* gene, total 17 somatic mutations, which located across 7 different exons (exon 1, 2, 3, 5, 6, 7 and 8), were identified in 15 of the 313 patients (4.8%), and no recurrent mutations were found (S2 Table). Of these 17 mutations, 8 located in the phosphatase domain and 9 located in the conserved domain 2 (C2) (Fig 1B), including 8 nonsense mutations, 5 missense mutations, 3 frameshift indels and 1 splice site mutation. Notably, two patients harbored two mutations in *PTEN* (Patient 124 harboring p.Q17X and p.C211X, Patient 258 harboring p.Y27N and p.G165R) (S2 Table). Three novel somatic mutations (p.K62X, p.N340IfsTer4 and c.635-12_636delTTAACCATGCAGAT) were detected in *PTEN*, all of which may lead to a truncated or non-functional PTEN protein. In addition, two germline mutations were found in *PTEN* (S3 Table). The mutation p.C136R was recorded in NCBI ClinVar database as pathogenic. Another germline mutation p.Q110E was novel and predicted to be tolerated by SIFT and benign by PolyPhen-2.

In *TP53* gene, total 84 somatic mutations were identified in 106 of the 313 patients (33.9%), with 5 patients harboring two mutations. All the somatic coding mutations of *TP53* located in exon 4, 5, 6, 7, 8, 9 and 11, and additional 3 splicing variants located in intron 4 and 9 (Fig 1G–1J and S2 Table). A large proportion of somatic mutations found in *TP53* clustered in the region from exon 4 to exon 8 within the DNA-binding domain, mutations of which presented in 97 cases (97/106, 91.5%). The somatic mutation of *TP53* included 62 missense mutations, 10 indels (5 inframe and 5 frameshift), 9 nonsense mutations and 3 splicing variants. Notably, 5 novel somatic mutations (p.Q136AfsTer5, p.K139_P142del, p.Y234dup, p.V274LfsTer31 and p.N310TfsTer35) were detected in *TP53*. All of these were frameshift mutations which may lead to deleterious effect on TP53 protein function. Additionally, 3 germline mutations were detected in *TP53* (S4 Table). The two missense mutations, p.G244S and p.P295L, were recorded in NCBI ClinVar as likely pathogenic and uncertain significance, respectively. The remaining one splicing variant c.559+1G>A was classified as pathogenic mutations.

### Multiple-gene and recurrent mutations in *AKT1*, *PIK3CA*, *PTEN* and *TP53*

Among the 190 somatic mutation carriers, 137 (137/190, 72.1%) harbored mutation in single gene (Table 2 and S3 Table). Total 51 patients (51/190, 26.8%) harbored co-mutation in two genes and 2 patients (2/190, 1.1%) harbored co-mutation in three genes. These included 2 patients (2/313, 0.6%) with co-mutations in *AKT1*-*PIK3CA*, 2 patients (2/313, 0.6%) with co-mutations in *AKT1-TP53*, 4 patients (4/313, 1.3%) with co-mutations in *PIK3CA-PTEN*, 40 patients (40/313, 12.8%) with co-mutations in *PIK3CA-TP53*, 3 patients (3/313, 1.0%) with co-mutations in *PTEN-TP53* and 2 patients (2/313, 0.6%) with co-mutations in *PIK3CA*-*PTEN*-*TP53*. No concurred mutation was observed in *AKT1*-*PTEN* and *AKT1*-*PIK3CA*-*TP53* genes (Table 2 and S2 Table).

Total 25 recurrent somatic mutations were found in this study (S2 Table). Among them, 10 mutations each recurred in >1% cases of this cohort of 313 patients (Table 4), including 1 mutation in *AKT1* (p.E17K), 5 mutations in *PIK3CA* (p.N345K, p.E542K, p.E545K, p.H1047R and p.H1047L) and 4 mutations in *TP53* (p.A159V, p.R175H, p.R213X and p.R248W). Overall, 125 of the 313 (39.9%) patients harbored at least one of these 10 mutations accounting for 55.7% of all mutations found in *AKT1*, *PIK3CA* and *TP53*. We did not observe any recurrent mutation in *PTEN* gene in this study. All of the *PTEN* mutations only presented in one patient each.

### Association of somatic mutations with clinicopathological characteristics

We analyzed correlations between somatic mutation status of the 4 genes and patient clinicopathological characteristics (Table 5). Comparing mutation carriers and non-carriers, *PIK3CA* mutation carriers were significantly more likely to be ER-positive (*P* = 0.041), PR-positive (*P* = 0.004) and invasive ductal carcinoma (IDC) (*P* = 0.002). *TP53* mutation carriers had a significant higher proportion of patients to be ER-negative (*P*<0.001), PR-negative (*P*<0.001), HER2-positive (*P* = 0.002), IHC p53 mutation positive (*P* = 0.018) and with high Ki67 expression (*P*<0.001) than non-carriers. No significant difference of clinicopathological characteristics was identified between mutation carriers and non-carriers of *AKT1* or *PTEN* (Table 4).

**Table 5:** Clinicopathological characteristics and associations with somatic mutation status in 313 breast cancer patients.

	Total	*AKT1* mutation (N = 10)			*PIK3CA* mutation (N = 114)			*PTEN* mutation (N = 15)			*TP53* mutation (N = 106)		
Characteristic	N (%)	Mutant (%)	WT (%)	P^a^	Mutant (%)	WT (%)	P^b^	Mutant (%)	WT (%)	P^c^	Mutant (%)	WT (%)	P ^d^
Age at diagnosis				0.900			0.202			0.127			0.311
≤35	14 (4.5)	1 (10.0)	13 (4.3)		4 (3.5)	10 (5.0)		0 (0.0)	14 (4.7)		6 (5.7)	8 (3.9)	
35–50	131 (41.8)	2 (20.0)	129 (42.6)		48 (42.1)	83 (41.7)		11 (73.3)	120 (40.3)		37 (34.9)	94 (45.4)	
≥50	168 (53.7)	7 (70.0)	161 (53.1)		62 (54.4)	106 (53.3)		4 (26.7)	164 (55.0)		63 (59.4)	105 (50.7)	
Mean ± SD	51.24 (±9.93)	51.10 (±13.98)	51.60 (±9.47)		52.84 (±10.33)	50.45 (±10.08)		48.33 (±7.85)	51.66 (±9.40)		51.68 (±9.41)	51.01 (±10.21)	
Lymph node metastasis				0.534			0.726			0.712			0.084
Positive (+)	137 (43.8)	3 (30.0)	134 (44.2)		51 (44.8)	86 (43.2)		6 (40.0)	131 (44.0)		54 (51.0)	83 (40.1)	
Negative (-)	170 (54.3)	7 (70.0)	163 (53.8)		60 (52.6)	110 (55.3)		9 (60.0)	161 (54.0)		51 (48.1)	119 (57.5)	
unknown	6 (1.9)	0 (0.0)	6 (2.0)		3 (2.6)	3 (1.5)		0 (0.0)	6 (2.0)		1 (0.9)	5 (2.4)	
ER status				0.326			**0.041**			0.629			**<0.001**
Positive (+)	222 (70.9)	9 (90.0)	213 (70.3)		89 (78.1)	133 (66.8)		12 (80.0)	210 (70.5)		55 (51.9)	167 (80.7)	
Negative (-)	90 (28.8)	1 (10.0)	89 (29.4)		25 (21.9)	65 (32.6)		3 (20.0)	87 (29.2)		51 (48.1)	39 (18.8)	
unknown	1 (0.3)	0 (0.0)	1 (0.3)		0 (0.0)	1 (0.5)		0 (0.0)	1 (0.3)		0 (0.0)	1 (0.5)	
PR status				0.868			**0.004**			0.546			**<0.001**
Positive (+)	163 (52.1)	6 (60.0)	157 (51.8)		72 (63.2)	91 (45.7)		9 (60.0)	154 (51.7)		35 (33.0)	128 (61.8)	
Negative (-)	148 (47.3)	4 (40.0)	144 (47.5)		42 (36.8)	106 (53.3)		6 (40.0)	142 (47.7)		71 (67.0)	77 (37.2)	
unknown	2 (0.6)	0 (0.0)	2 (0.7)		0 (0.0)	2 (1.0)		0 (0.0)	1 (0.3)		0 (0.0)	2 (1.0)	
HER2 status				0.277			0.194			0.086			**0.002**
Positive (+)	89 (28.4)	1 (10.0)	88 (29.0)		27 (23.7)	62 (31.2)		1 (6.7)	88 (29.5)		43 (40.6)	46 (22.2)	
Negative (-)	177 (56.6)	8 (80.0)	169 (55.8)		68 (59.6)	109 (54.7)		12 (80.0)	165 (55.4)		51 (48.1)	126 (60.9)	
unknown	47 (15.0)	1 (10.0)	46 (15.2)		19 (16.7)	28 (14.1)		2 (13.3)	45 (15.1)		12 (11.3)	35 (16.9)	
*BRCA* status				1.000			**0.008**			1.000			**0.001**
*BRCA1* (+)	5 (1.6)	0 (0.0)	5 (1.6)		1 (0.9)	4 (2.0)		0 (0.0)	5 (1.7)		5 (4.7)	0 (0.0)	
*BRCA2* (+)	12 (3.8)	0 (0.0)	12 (4.0)		0 (0.0)	12 (6.0)		0 (0.0)	12 (4.0)		1 (1.0)	11 (5.3)	
*BRCA1/2* (-)	296 (94.6)	10 (100.0)	286 (94.4)		113 (99.1)	183 (92.0)		15 (100.0)	281 (94.3)		100 (94.3)	196 (94.7)	
p53 mutation (IHC)				0.630			0.060			0.130			**0.018**
Positive (+)	239 (76.4)	9 (90.0)	230 (75.9)		94 (82.5)	145 (72.9)		9 (60.0)	230 (77.2)		90 (84.9)	149 (72.0)	
Negative (-)	64 (20.4)	1 (10.0)	63 (20.8)		17 (14.9)	47 (23.6)		6 (40.0)	58 (19.5)		14 (13.2)	50 (24.1)	
unknown	10 (3.2)	0 (0.0)	10 (3.3)		3 (2.6)	7 (3.5)		0 (0.0)	10 (3.4)		2 (1.9)	8 (3.9)	
Ki67-expression				0.901			0.941			0.774			**<0.001**
<14%	134 (42.8)	5 (50.0)	129 (42.6)		49 (43.0)	85 (42.7)		7 (46.7)	127 (42.6)		24 (22.6)	110 (53.1)	
≥14%	177 (56.6)	5 (50.0)	172 (56.8)		64 (56.1)	113 (56.8)		8 (53.3)	169 (56.7)		82 (77.4)	95 (45.9)	
unknown	2 (0.6)	0 (0.0)	2 (0.6)		1 (0.9)	1 (0.5)		0 (0.0)	2 (0.7)		0 (0.0)	2 (1.0)	
Tumor grade				1.000			0.487			1.000			0.051
I	11 (3.5)	0 (0.0)	11 (3.6)		4 (3.5)	7 (3.5)		0 (0.0)	11 (3.7)		2 (1.9)	9 (4.3)	
II	247 (78.9)	8 (80.0)	239 (78.9)		99 (86.8)	148 (74.4)		12 (80.0)	235 (78.9)		83 (78.3)	164 (79.2)	
III or IV	19 (6.1)	0 (0.0)	19 (6.3)		5 (4.4)	14 (7.0)		0 (0.0)	19 (6.4)		11 (10.4)	8 (3.9)	
Unknown	36 (11.5)	2 (20.0)	34 (11.2)		6 (5.3)	30 (15.1)		3 (20.0)	33 (11.1)		10 (9.4)	26 (12.6)	
Tumor type				0.259			**0.002**			0.054			0.311
IDC	221 (70.6)	5 (50.0)	116 (38.3)		94 (82.4)	127 (63.8)		7 (46.7)	214 (71.8)		75 (70.7)	146 (70.5)	
ILC	5 (1.6)	1 (10.0)	4 (1.3)		1 (0.9)	4 (2.0)		1 (6.7)	4 (1.3)		0 (0.0)	5 (2.4)	
Others	87 (27.8)	4 (40.0)	83 (27.4)		19 (16.7)	68 (34.2)		7 (46.7)	80 (26.8)		31 (29.3)	56 (27.1)	
Molecular subtype				0.484			0.111			0.467			**<0.001**
Luminal A	86 (27.5)	5 (50.0)	81 (26.7)		35 (30.7)	51 (25.6)		5 (33.3)	81 (27.2)		10 (9.4)	76 (36.7)	
Luminal B	128 (40.9)	4 (40.0)	124 (40.9)		49 (43.0)	79 (39.7)		7 (46.7)	121 (40.6)		48 (45.3)	80 (38.6)	
Basal-like	40 (12.8)	0 (0.0)	40 (13.2)		13 (11.4)	27 (13.6)		3 (20.0)	37 (12.4)		25 (23.6)	15 (7.3)	
HER2-enriched	37 (11.8)	1 (10.0)	36 (11.9)		7 (6.1)	30 (15.1)		0 (0.0)	37 (12.4)		20 (18.9)	17 (8.2)	
Unknown	22 (7.0)	0 (0.0)	22 (7.3)		10 (8.8)	12 (6.0)		0 (0.0)	22 (7.4)		3 (2.8)	19 (9.2)	

ER, estrogen receptor; PR, progesterone receptor; HER2, human epidermal growth factor receptor 2.

IDC, invasive ductal carcinoma; ILC, invasive lobular carcinoma; IHC, immunohistochemistry; SE, standard error.

^a^ *AKT1* mutation carriers versus *AKT1* mutation non-carriers

^b^ *PIK3CA* mutation carriers versus *PIK3CA* mutation non-carriers

^c^ *PTEN* mutation carriers versus *PTEN* mutation non-carriers

^d^ *TP53* mutation carriers versus *TP53* mutation non-carriers.

Here, *P* values for comparing difference of age were calculated by the Wilcoxon Rank Sum test; while *P* values for comparing categorical variables across other clinicopathological characteristics were calculated by χ^2^ test; *P* value<0.05 in bold.

Furthermore, we assessed whether these somatic mutations were associated with deleterious germline *BRCA1*/*2* mutations. All of the 313 patients have been tested for *BRCA1*/*2* mutations by NGS in our previous study [9]. As shown in Table 5, almost all of the *AKT1*, *PIK3CA* and *PTEN* somatic mutation carriers did not harbor *BRCA1/2* mutation, except that one *PIK3CA* somatic mutation carrier had a *BRCA1* mutation. Five *TP53* somatic mutation carriers co-harbored *BRCA1* mutation and one *TP53* somatic mutation carrier co-harbored *BRCA2* mutation. Notably, all of the five *BRCA1* mutation positive patients harbored *TP53* somatic mutations (*P* = 0.001).

### Somatic mutations distribution across different molecular subtypes

**Fig 2:**
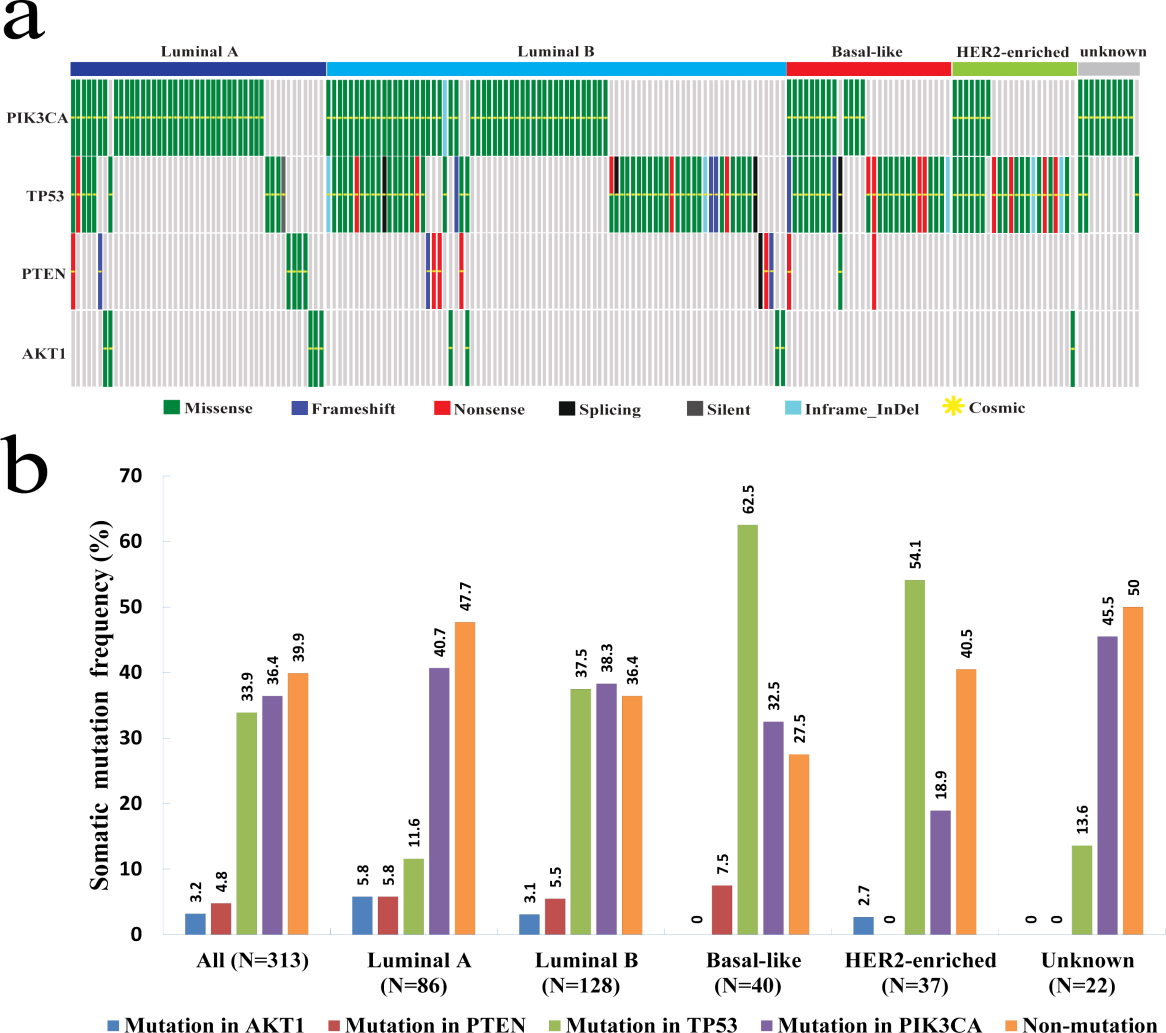
The distribution of somatic mutations in different breast cancer subtypes of the 313 breast cancer patients. a. The graphical summary of somatic mutations of the 4 genes in molecular subtypes. All of the 190 tumor samples with 4 gene somatic mutations are grouped into 5 groups: luminal A (n = 46), luminal B (n = 81), basal-like (n = 29), HER2-enriched (n = 22) and unknown (n = 11). The stripe panel shows every specific case harboring the 4 gene mutation with different mutation types. One stripe indicates one patient. Green stripe indicates missense mutation; Blue stripe indicates frameshift mutation; Red stripe indicates nonsense mutation; Black stripe indicates split site mutation; Dark Gray stripe indicates silent mutation; Cyan stripe indicates inframe indel mutation; Yellow star indicates mutation recorded in COSMIC database. b. The somatic mutation frequency of *AKT1*, *PTEN*, *PIK3CA* and *TP53* in different breast cancer subtypes. The frequency of somatic mutations for individual gene is shown in the bar chart in various groups according to molecular subtypes of breast cancer. These groups include All (N = 313), luminal A (N = 86), luminal B (N = 128), basal-like (N = 40), HER2-enriched (N = 37) and unknown (N = 22).

The distribution of the somatic mutations of the 4 genes varied in different breast cancer molecular subtypes (Fig 2). *PIK3CA* mutations occurred at high frequency in luminal A (40.7%) and luminal B (38.3%) tumors, while relatively low in basal-like (32.5%) and HER2-enriched (18.9%) tumors (Fig 2). In contrast, *TP53* mutations were more common in basal-like (62.5%) and HER2-enriched (54.1%) tumors than in luminal A (11.6%) and luminal B (37.5%) tumors (Fig 2). *AKT1* mutations only occurred in luminal A (5.8%), luminal B (3.1%) and HER2-enriched (2.7%) tumors. *PTEN* mutations only occurred in luminal A (5.8%), luminal B (5.5%) and basal-like (7.5%) tumors. The associations between somatic mutation of the 4 genes and breast cancer molecular subtypes were analyzed (Table 5). The *TP53* mutations showed significant association with breast cancer subtypes (*P*<0.001) and had higher proportion of patients with basal-like (23.6% vs. 7.3%) and HER2-enriched (18.9% vs. 8.2%) tumors, comparing with non-*TP53* mutations.

## Discussion

In this study, by integrating microfluidic PCR-based target enrichment and NGS technologies, we sequenced the entire coding regions and exon-intron boundaries of *TP53* and three PI3K pathway genes (*AKT1*, *PIK3CA*, *PTEN*) in paired tumor and normal tissue samples from 313 Chinese breast cancer patients. Our results showed that somatic mutations of these genes occurred at high frequency among Chinese breast cancer patients. Previously, several studies have conducted mutational analysis including *AKT1*, *PIK3CA*, *PTEN* and/or *TP53* genes in breast cancer worldwide [6, 25–42]. However, few studies have focused on the comprehensive study of *AKT1*, *PIK3CA*, *PTEN* and *TP53* mutations altogether in Chinese breast cancer patients. Most of these studies focused on either selected hotspot sites or selected exons of these four genes [37–43]. As shown in S4 Table, due to the differences of detection methods and studied regions, the reported mutation frequency of these four genes varied a lot among different studies and different populations. The mutation frequencies of *AKT1*, *PIK3CA*, *PTEN*, and *TP53* in Chinese population were reported to range 0–4.4%, 7.5–38.8%, 0–4.8% and 10.0–33.9% respectively (S4 Table). In other populations, these frequencies were reported to range 1.4–6.0%, 7.1–45.0%, 1.0–5% and 27.2–38.8% respectively (S4 Table). In all studies, *PIK3CA* and *TP53* were consistently the top two frequently mutated genes, which confirmed their important role in breast carcinogenesis.

In addition to high frequency of *PIK3CA* and *TP53* single-gene somatic mutation, *TP53-PIK3CA* co-mutations were detected as high as 12.8% in our cohort, compared that as 8.7% in a TCGA cohort [23] (Table 2). This co-occurrence pattern was also discovered by prior studies with frequency as 5.3% in 120 breast cancer patients [28] and as 5.9% in 1766 breast cancer patients [44]. Previous *in vivo* study has confirmed that *TP53* and *PIK3CA* mutations show cooperation in mammary tumor formation in mice [45]. It have been reported that *TP53*-*PIK3CA* co-mutation carriers had worst disease-free survival comparing with non-mutation carriers, *PIK3CA*-mutation-only or *TP53*-mutation-only carriers [46]. Since a high frequency of *TP53*-*PIK3CA* co-mutations was detected in our cohort, this mutation pattern need to be evaluated closely in clinical settings for Chinese breast cancer patient in the future.

Cancer hotspot mutations carry valuable information for diagnosis, prognosis and treatment [47]. In this cohort, total 10 mutations were found to be recurrently mutated in >1% patients accounting for 55.7% somatic mutations in *AKT1*, *PIK3CA* and *TP53* (Table 4). Of these 10 mutations, *AKT1* p.E17K, three *PIK3CA* mutations within the PI3Ka (E542K and E545K) and PI3Kc (H1047R) domains and two *TP53* mutations (p.R175H and p.R248W) within the DNA binding domain were well established hotspots in breast cancer [48, 49]. Additional 3 mutations (p.N345K and p.H1047L in *PIK3CA*, p.R213X in *TP53*) were also reported as hotspots by a recent study on a large number of tumors by a novel statistical algorithm [50]. The p.H1047L mutation occurred at the same location as p.H1047R, which were also detected by a study on Chinese breast cancer patients [42]. The mutation *TP53* p.A159V was also detected by another study on breast cancer with the frequency as 0.9% (5/560) [5]. These hotspot mutations may be important candidate target for clinical applications in cancer treatment and screening.

Previous studies suggested that somatic mutation was one of the mechanisms leading to PTEN loss [51, 52]. In this study, the frequency of somatic mutations of *PTEN* was reported as 4.8%, while loss of *PTEN* in protein expression was reported as high as 48% in breast cancer [53]. The reason was that other mechanisms such as promoter methylation, loss of heterozygosity, transcriptional or post-transcriptional regulation can also lead to PTEN loss. In this study, 11 out of the 17 somatic mutations found in *PTEN* were stopgain SNVs or frameshift indels which can cause truncated PTEN protein. And the other 6 *PTEN* somatic mutations were predicted to be deleterious or probably damaging (S2 Table). Taken together, all *PTEN* somatic mutations may lead to deleterious effect on protein function, which suggested that PTEN alteration play a critical role in breast tumorgenesis.

AKT1 is a downstream mediator of phosphatidylinositol 3-kinase. In line with previous studies [39, 54] on Chinese breast cancer patients, we detected only one hotspot mutation (p.E17K) in the pleckstrin homology domain. Recently, it has been demonstrated that mutation *AKT1* p.E17K is a therapeutic target which is sensitive to AKT inhibitors in breast cancer patients [55]. Thus 9 out of the 10 (90%) *AKT1* somatic mutation carriers with p.E17K mutations in this study (S2 Table) may be good candidates for AKT inhibitors treatment.

In conclusion, our results showed that somatic mutations in *AKT1*, *PIK3CA*, *PTEN* and *TP53* genes were common events in Chinese breast cancer patients and had distinct spectrum across different breast cancer subtypes. Total 60.7% of the patients harbored at least 1 somatic mutation. *PIK3CA* somatic mutations were significantly associated with ER-positive or PR-positive tumors. *TP53* somatic mutations were significantly associated with ER-negative, PR-negative, HER2-positive, *BRCA1* mutation, Ki67 high expression and basal-like tumors. These findings provided a comprehensive mutational characterization of *AKT1*, *PIK3CA*, *PTEN* and *TP53* genes in Chinese breast cancer patients with valuable implications for clinical management and optimal design of clinical trials in the future.

## Supporting information

S1 Fig Verification of somatic mutations in tumor tissues by Sanger sequencing. (PDF)

S2 Fig Verification of germline mutations in tumor/normal tissues by Sanger sequencing. (PDF)

S1 Table The primer sequences of the *AKT1*, *PIK3CA*, *PTEN* and *TP53* genes. (XLSX)

S2 Table Somatic mutations of *AKT1*, *PIK3CA*, *PTEN* and *TP53* genes in the 313 breast cancer patients. (XLSX)

S3 Table Germline variants of *PIK3CA*, *PTEN* and *TP53* genes in the 313 breast cancer patients. (XLSX)

S4 Table Worldwide distribution pattern of *AKT1*, *PIK3CA*, *PTEN* and *TP53* mutations in primary breast cancer. (XLSX)
