# Supplementary material for: Prevalence and spectrum of AKT1, PIK3CA, PTEN and TP53 somatic mutations in Chinese breast cancer patients
Source: PLoS One. 2018 Sep 13;13(9):e0203495. doi: 10.1371/journal.pone.0203495 (PMC6136723; doi:10.1371/journal.pone.0203495)

SUPPLEMENTARY FIGURES

Figure S1. Verification of somatic mutations in tumor tissues by Sanger sequencing

AKT1

P46： AKT1 c.49G>A p.E17K

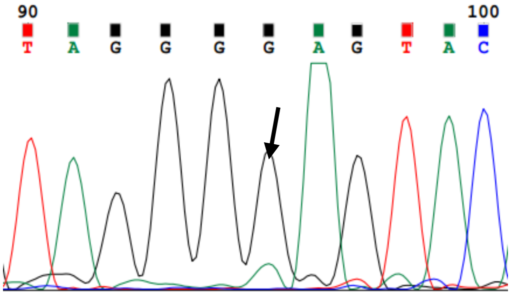

P108： AKT1 c.49G>A p.E17K

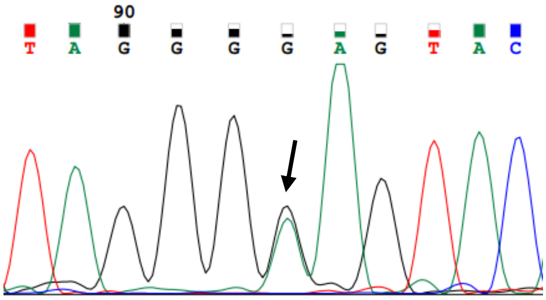

P155： AKT1 c.49G>A p.E17K

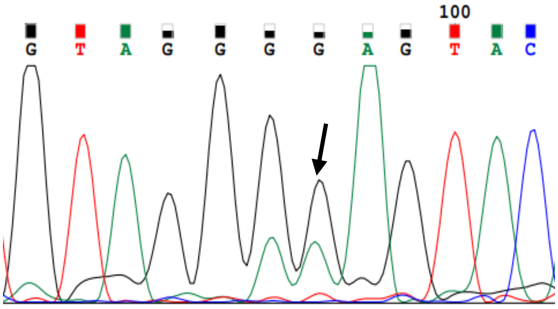

P242： AKT1 c.49G>A p.E17K

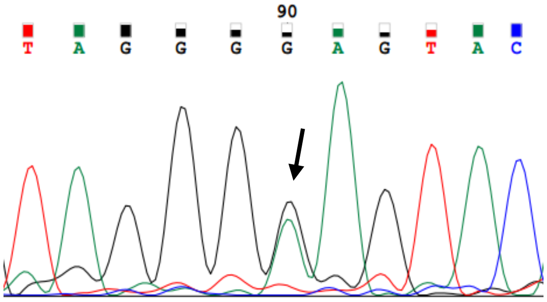

PIK3CA

P34: PIK3CA c.1035T>A p.N345K

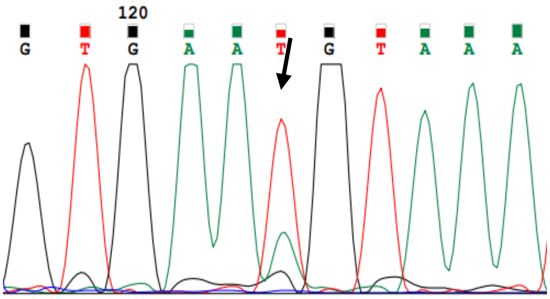

P142: PIK3CA c.1035T>A p.N345K

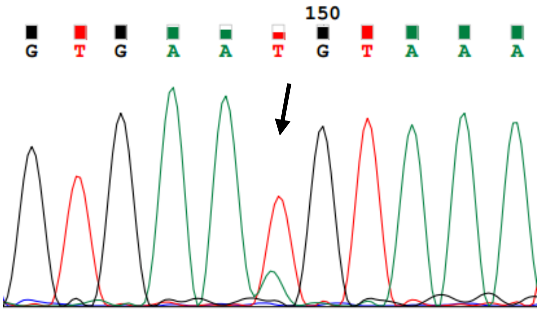

P186: PIK3CA c.1035T>A p.N345K

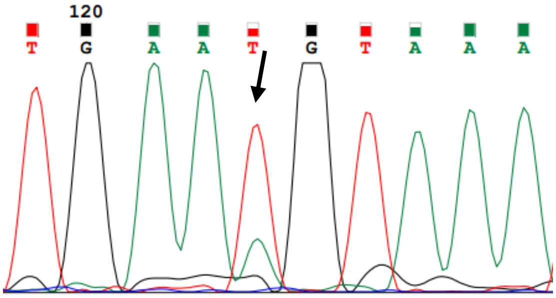

P149: PIK3CA c.1035T>A p.N345K

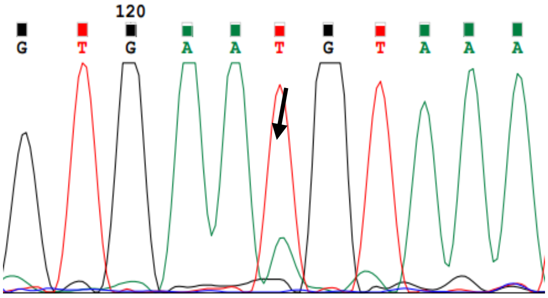

P216: PIK3CA c.1048G>C p.D350H

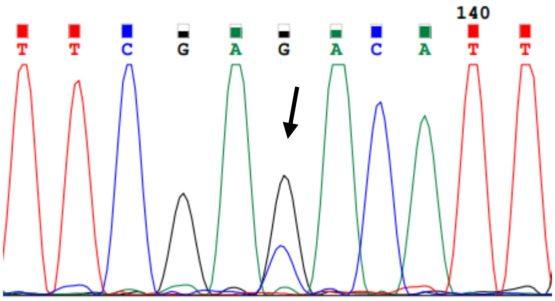

P9: PIK3CA c.1256_1261delACTGTC p.H419_C420del

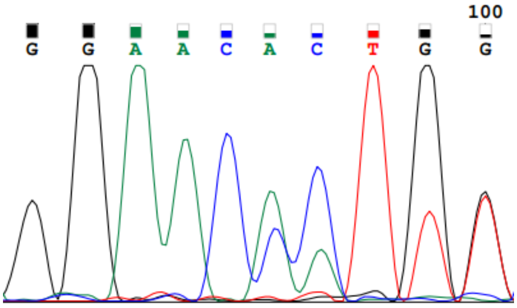

P111: PIK3CA c.1258T>C p.C420R

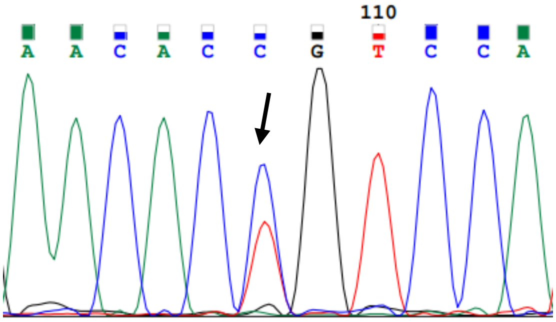

P270: PIK3CA c.1258T>C p.C420R

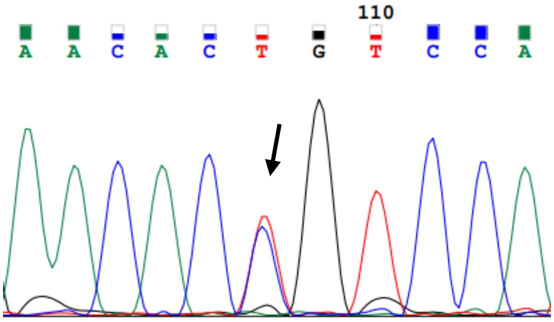

P124: PIK3CA c.1624G>A p.E542K

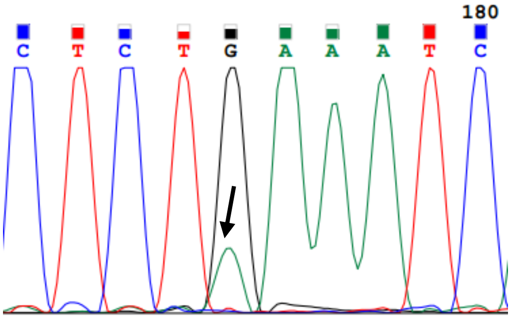

P207: PIK3CA c.1624G>A p.E542K

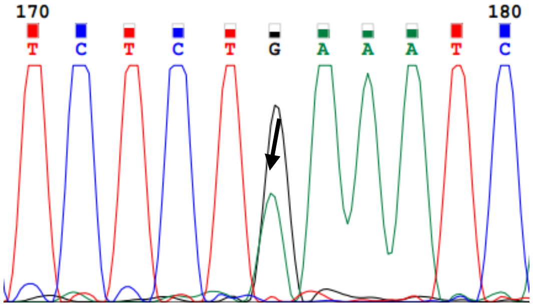

P24: PIK3CA c.1633G>A p.E545K

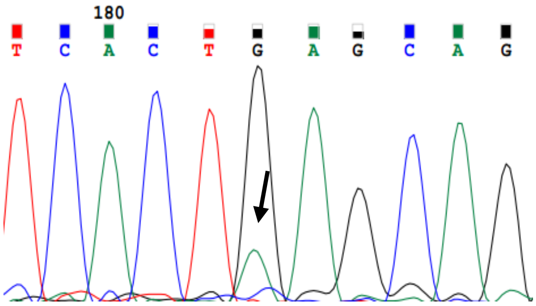

P35: PIK3CA c.1633G>A p.E545K

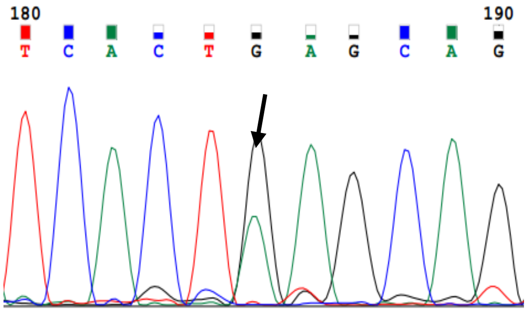

P44: PIK3CA c.1633G>A p.E545K

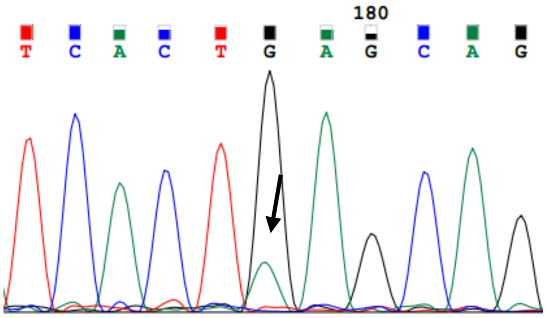

P56: PIK3CA c.1633G>A p.E545K

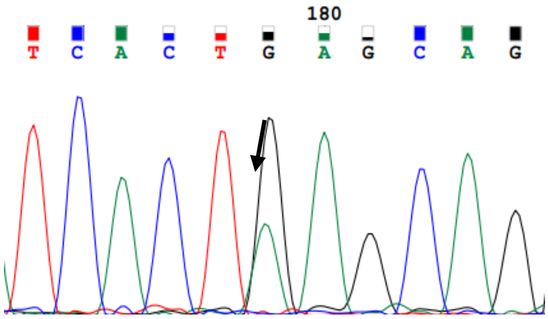

P58: PIK3CA c.1633G>A p.E545K

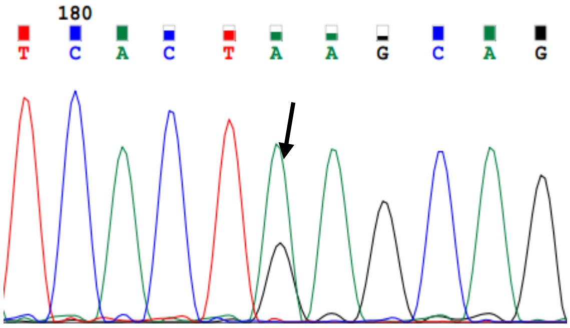

P69: PIK3CA c.1633G>A p.E545K

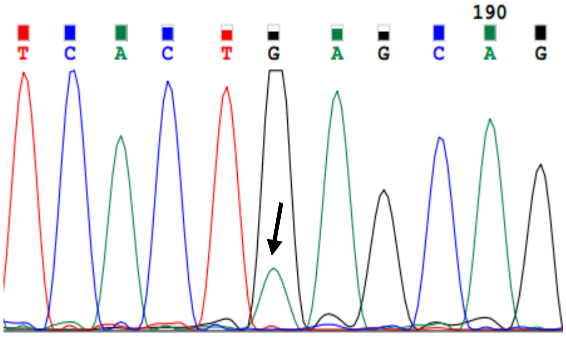

P177: PIK3CA c.1633G>A p.E545K

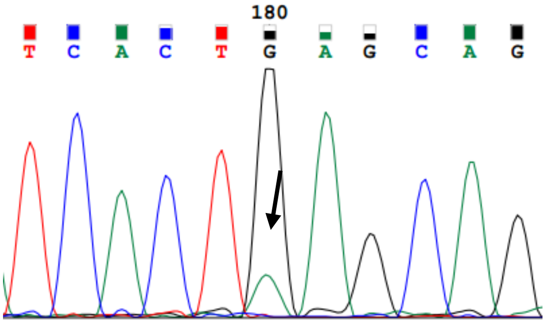

P210: PIK3CA c.1633G>A p.E545K

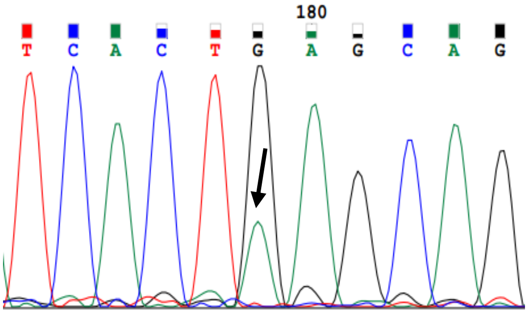

P232: PIK3CA c.1633G>A p.E545K

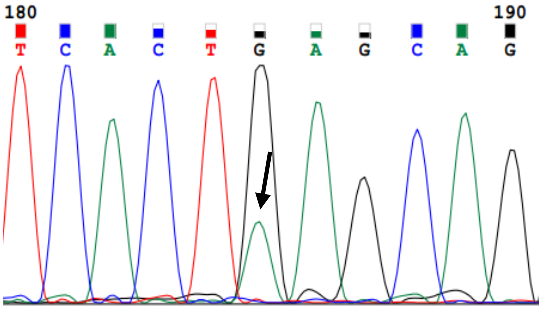

P247: PIK3CA c.1633G>A p.E545K

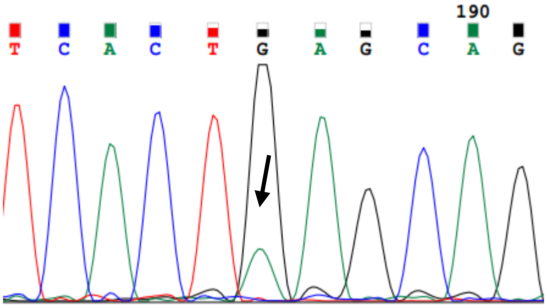

P300: PIK3CA c.1633G>A p.E545K

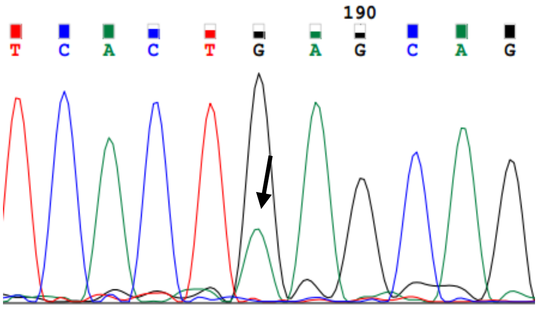

P66: PIK3CA c.3019G>C p.G1007R

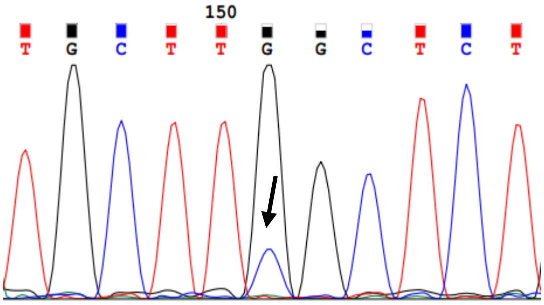

P6: PIK3CA c.3140A>G p.H1047R

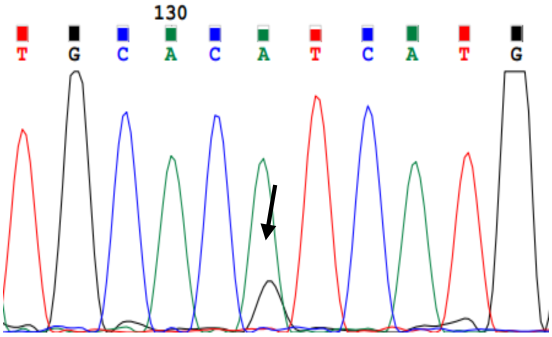

P8: PIK3CA c.3140A>G p.H1047R

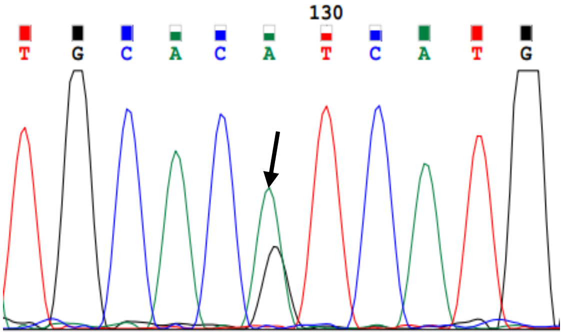

P19: PIK3CA c.3140A>G p.H1047R

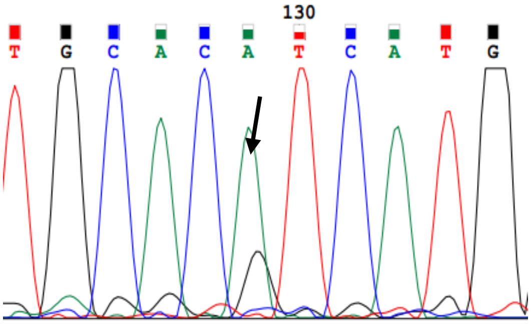

P40: PIK3CA c.3140A>G p.H1047R

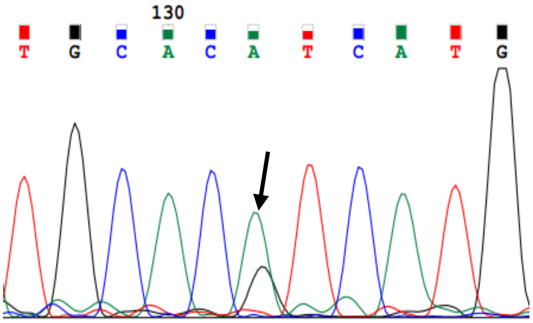

P57: PIK3CA c.3140A>G p.H1047R

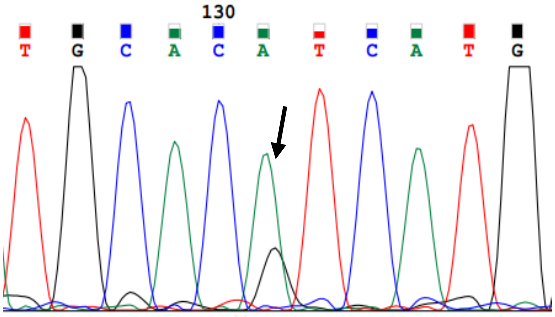

P109: PIK3CA c.3140A>G p.H1047R

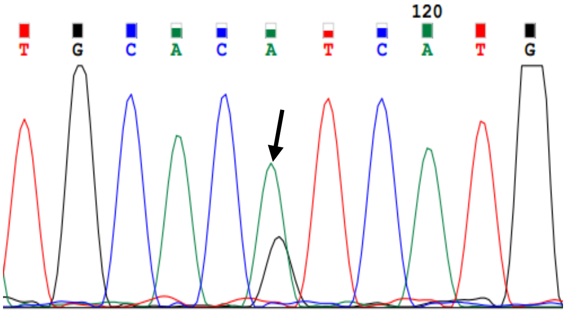

P112: PIK3CA c.3140A>G p.H1047R

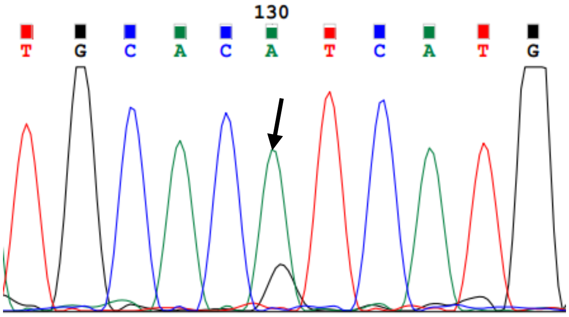

P115: PIK3CA c.3140A>G p.H1047R

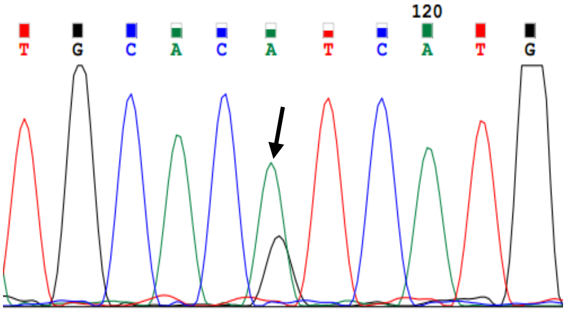

P116: PIK3CA c.3140A>G p.H1047R

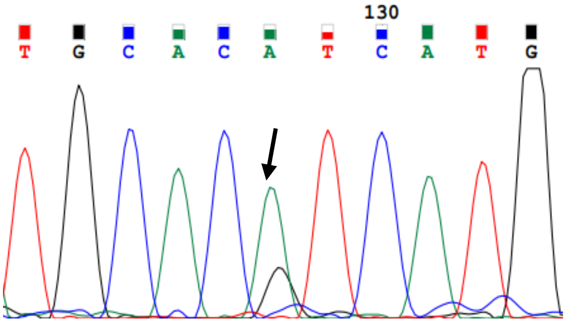

P134: PIK3CA c.3140A>G p.H1047R

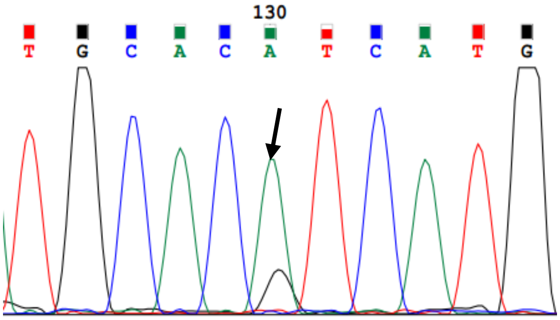

P140: PIK3CA c.3140A>G p.H1047R

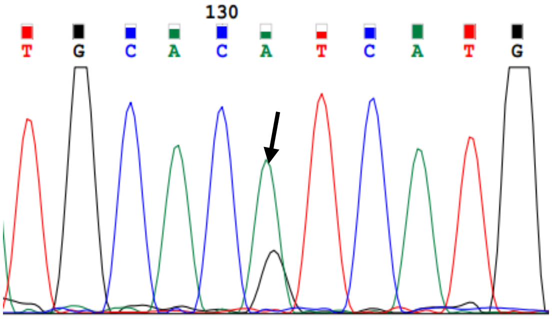

P144: PIK3CA c.3140A>G p.H1047R

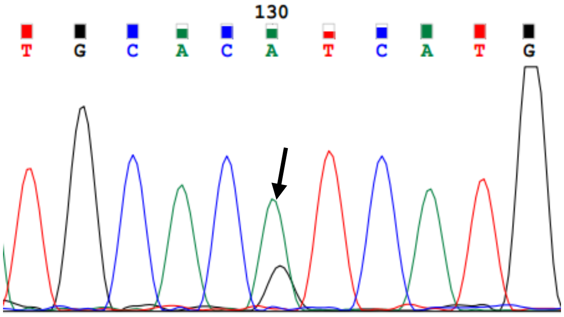

P145: PIK3CA c.3140A>G p.H1047R

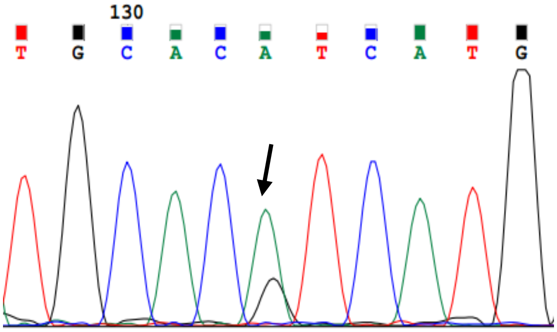

P191: PIK3CA c.3140A>G p.H1047R

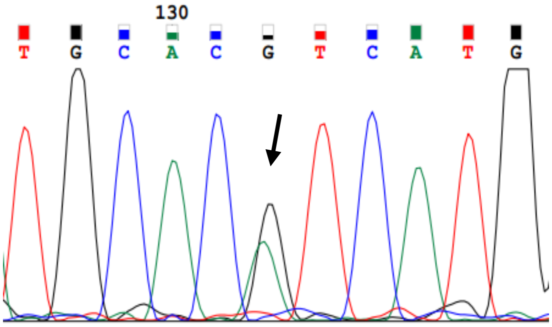

P192: PIK3CA c.3140A>G p.H1047R

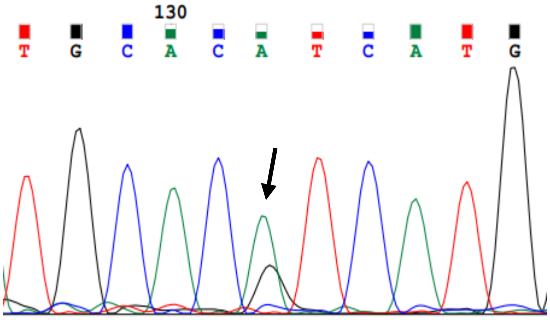

P203: PIK3CA c.3140A>G p.H1047R

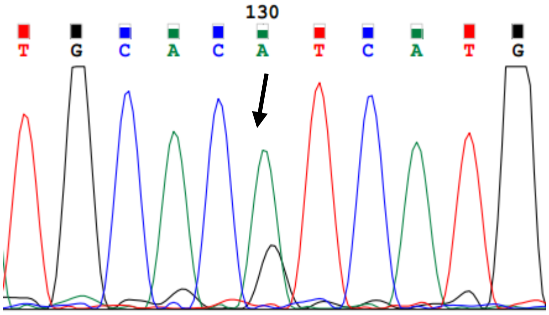

P213: PIK3CA c.3140A>G p.H1047R

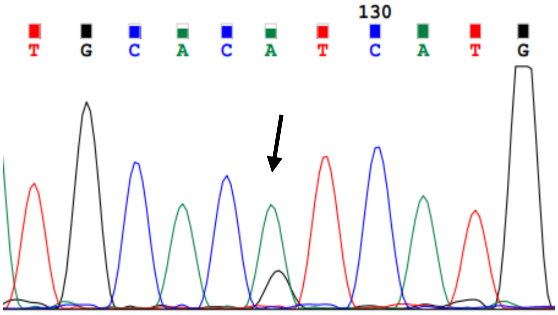

P218: PIK3CA c.3140A>G p.H1047R

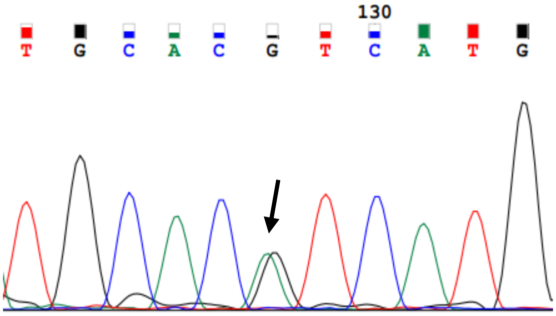

P261: PIK3CA c.3140A>G p.H1047R

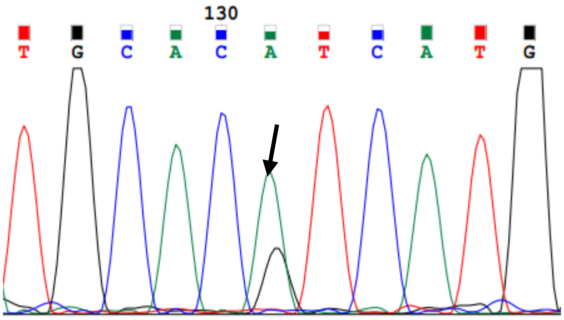

P293: PIK3CA c.3140A>G p.H1047R

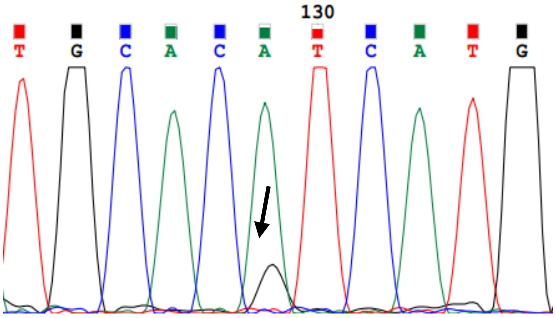

P304: PIK3CA c.3140A>G p.H1047R

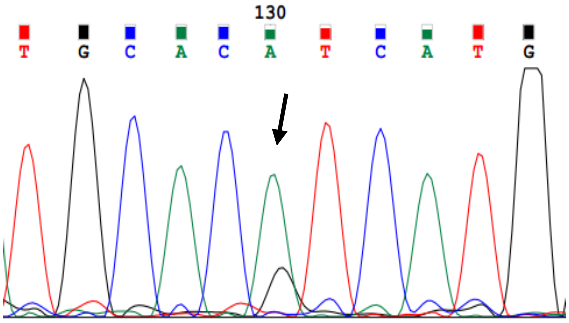

P307: PIK3CA c.3140A>G p.H1047R

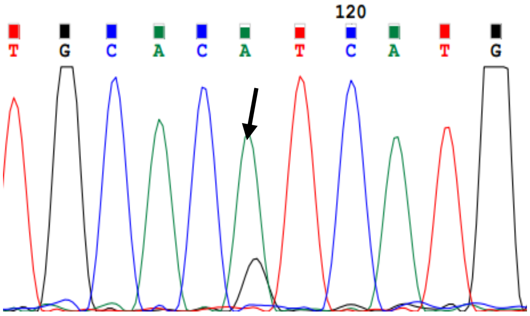

P91: PIK3CA c.3140A>T p.H1047L

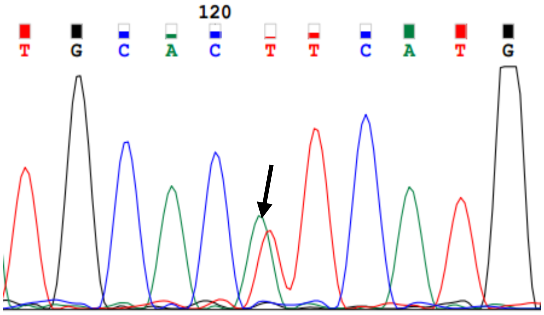

P155: PIK3CA c.3140A>T p.H1047L

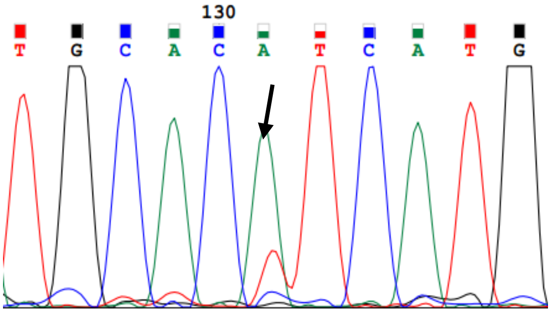

P170: PIK3CA c.3140A>T p.H1047L

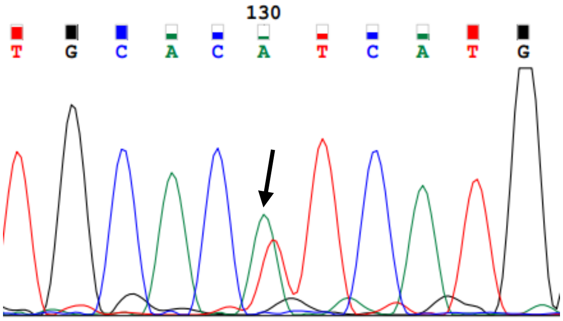

P175: PIK3CA c.3140A>T p.H1047L

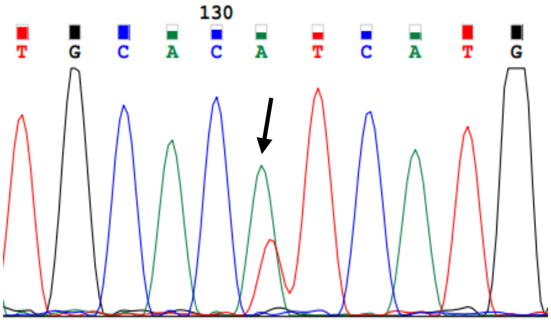

P208: PIK3CA c.3140A>T p.H1047L

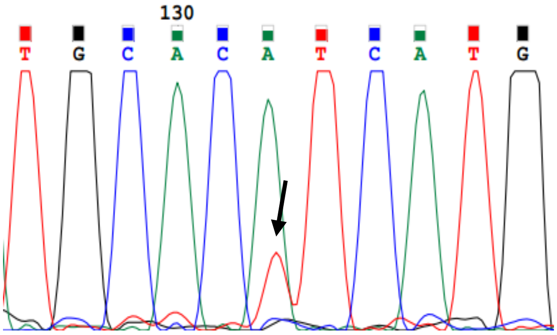

P266: PIK3CA c.3140A>T p.H1047L

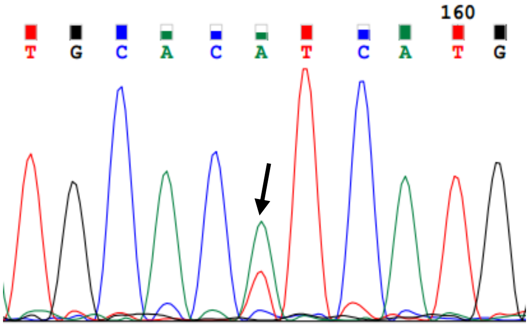

P288: PIK3CA c.3140A>T p.H1047L

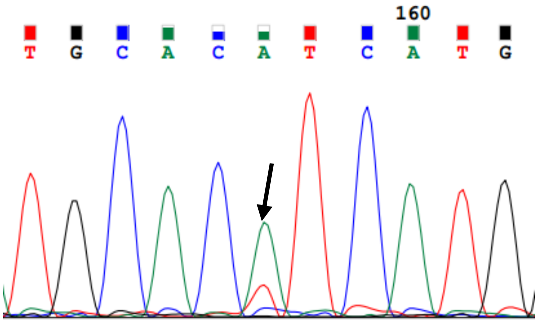

PTEN

P59: PTEN c.45_46insT p.Y16LfsTer28

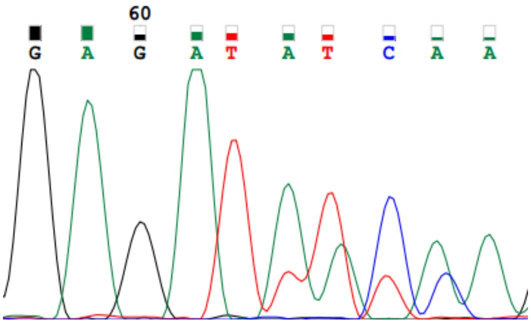

P124: PTEN c.49C>T p.Q17*

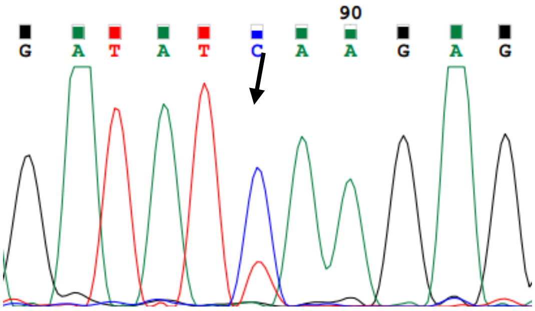

P258: PTEN c.79T>A p.Y27N

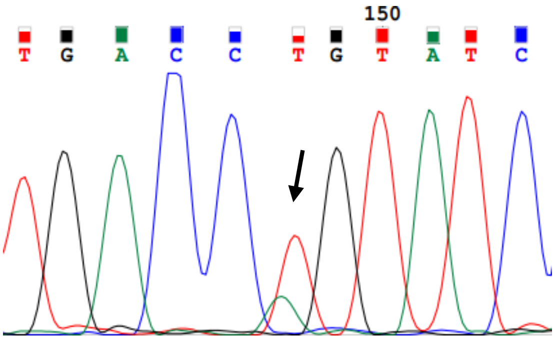

P209: PTEN c.113C>G p.P38R

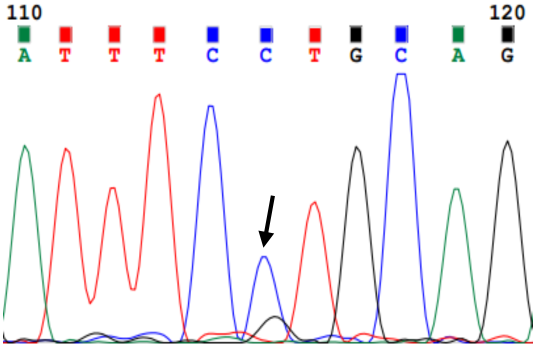

P82: PTEN c.184A>T p.K62*

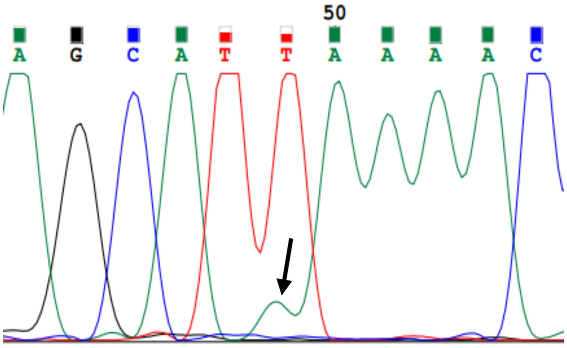

P135: PTEN c.389G>C p.R130P

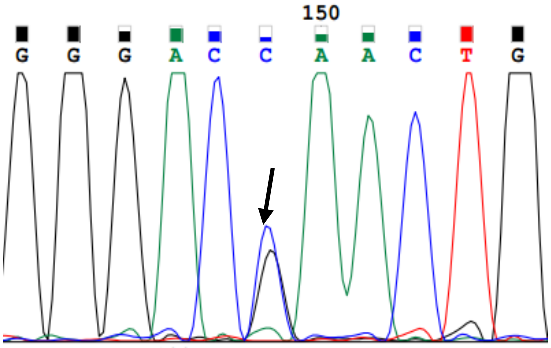

P54: PTEN c.635-12_636delTTAACCATGCAGAT

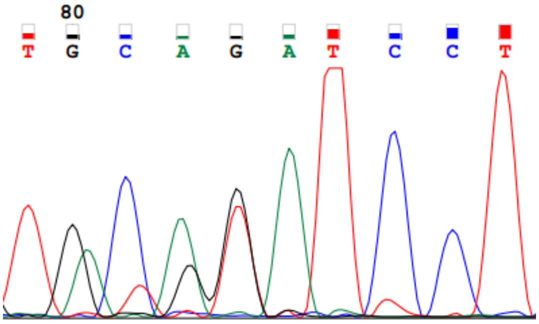

P189: PTEN c.697C>T p.R233*

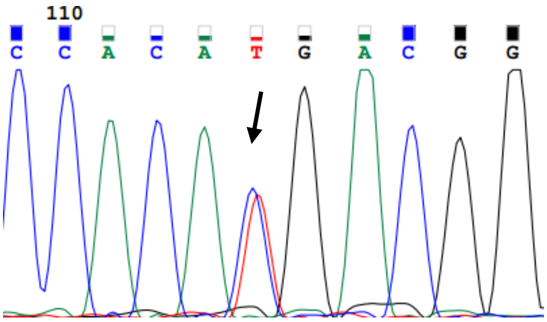

P191: PTEN c.892C>T p.Q298*

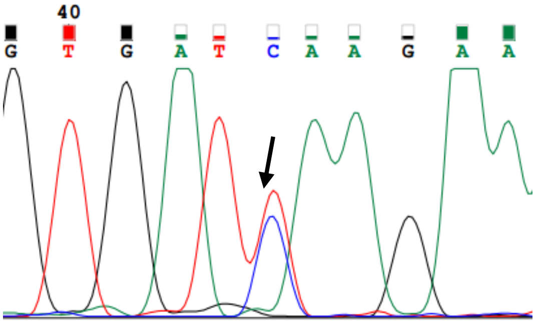

P308: PTEN c.1003C>T p.R335*

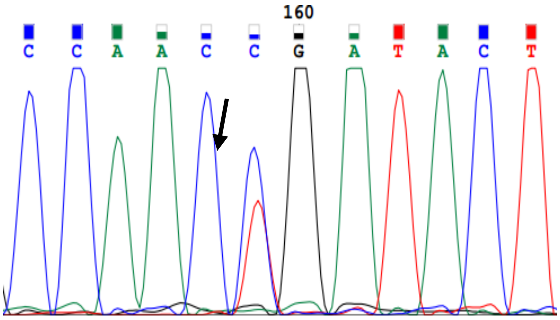

P207: PTEN c.955_958delACTT p.T319Ter

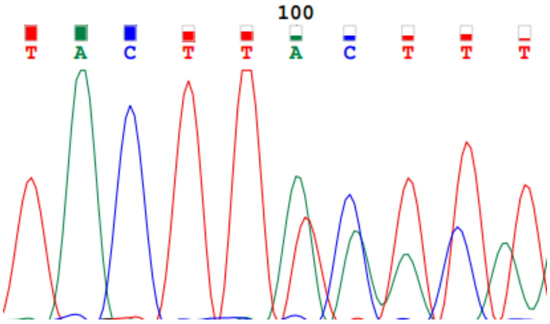

P224: PTEN c.1019delA p.N340IfsTer4

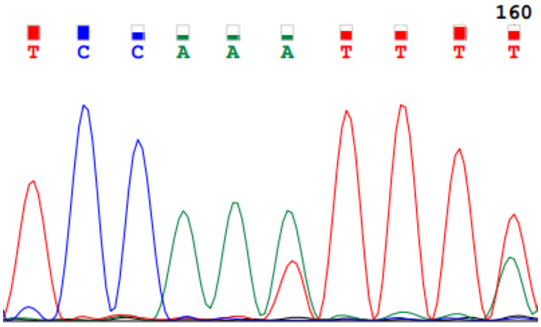

TP53

P183: TP53 c.166G>T p.E56*

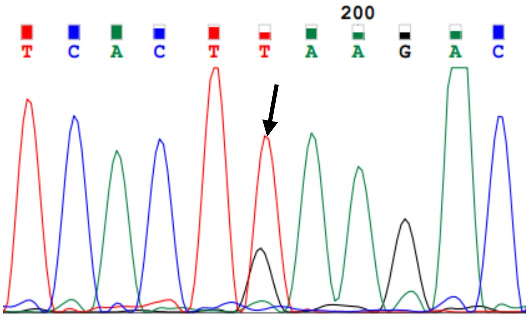

P82: TP53 c.281C>A p.S94*

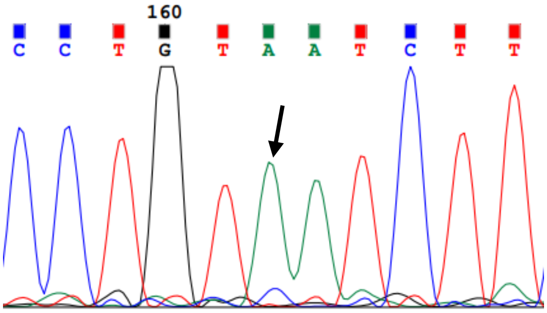

P223: TP53 c.329G>C p.R110P

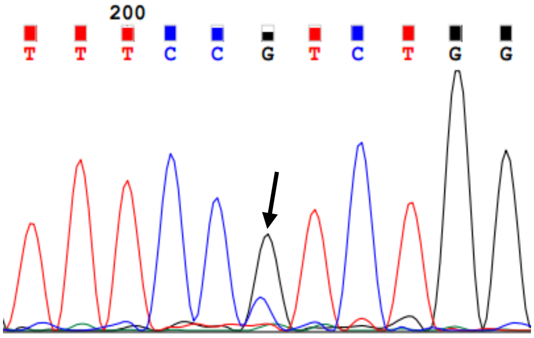

P265: TP53 c.338T>G p.F113C

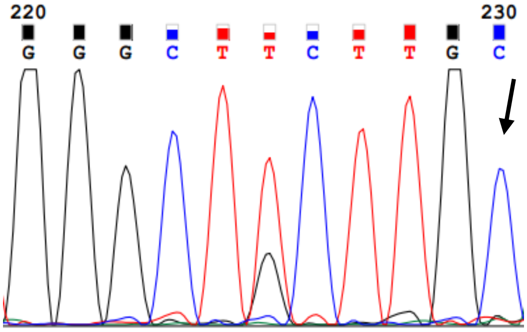

P72: TP53 c.375G>C p.T125T

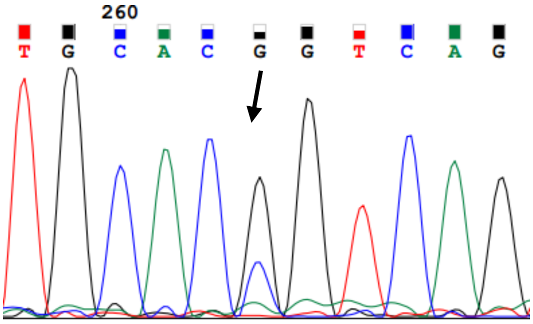

P270: TP53 c.376-2delA

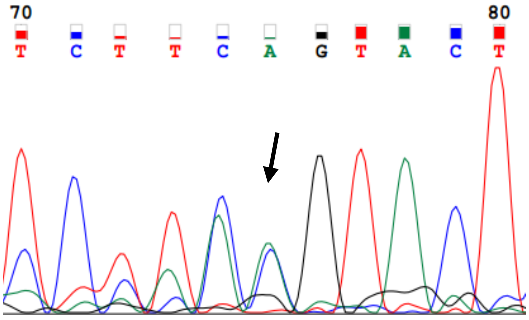

P101: TP53 c.396G>C p.K132N

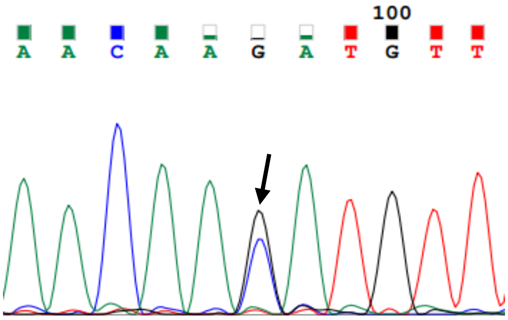

P218: TP53 c.406_428delCAACTGGCCAAGACCTGCCCTGT p.Q136AfsTer5

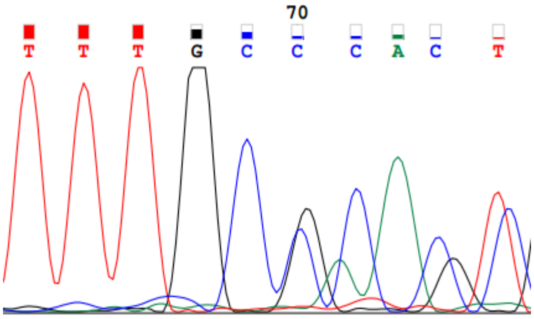

P39: TP53 c.414_425delCAAGACCTGCCC p.K139_P142del

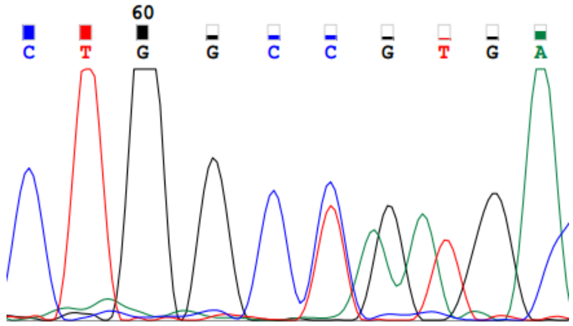

P175: TP53 c.423_425delCCC p.P142del

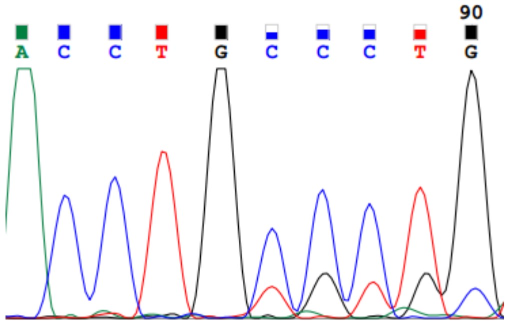

P27: TP53 c.428T>C p.V143A

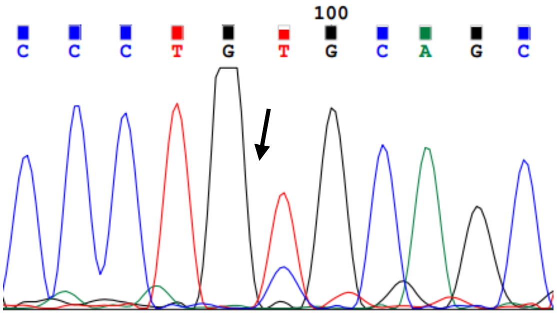

P81: TP53 c.431A>C p.Q144P

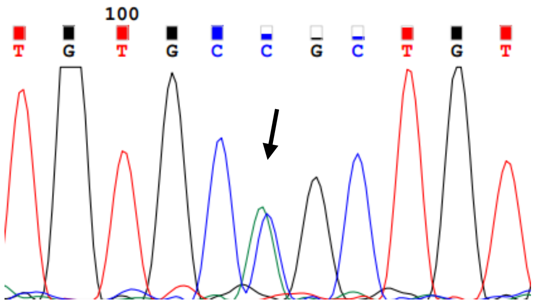

P166: TP53 c.469G>T p.V157F

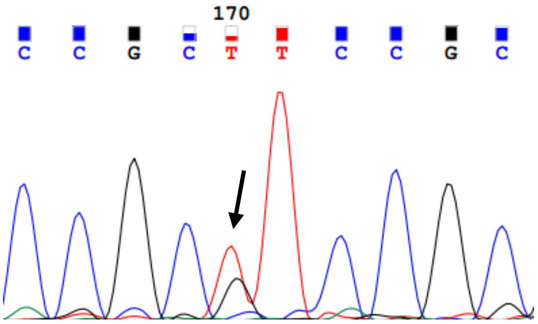

P116: TP53 c.475G>C p.A159P

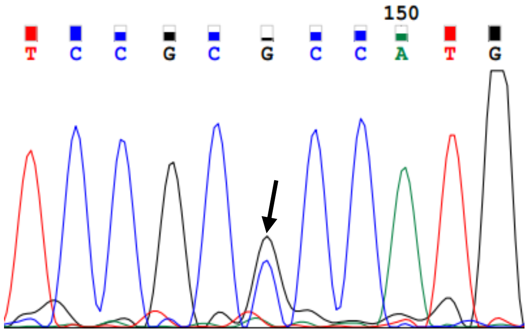

P9: TP53 c.476C>T p.A159V

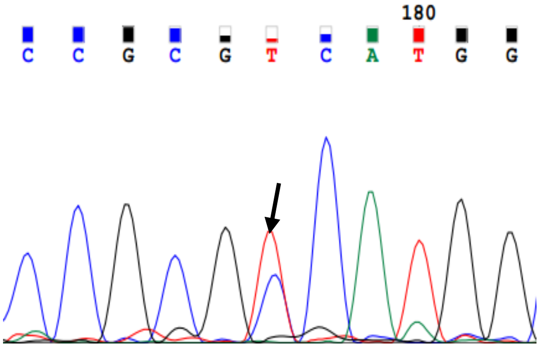

P102: TP53 c.485T>A p.I162N

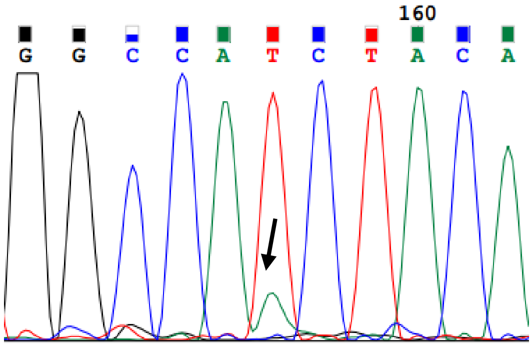

P28: TP53 c.488A>G p.Y163C

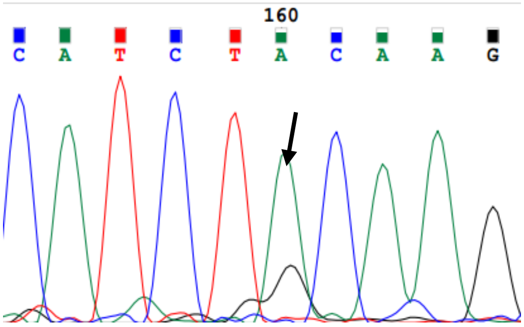

P65: TP53 c.517G>T p.V173L

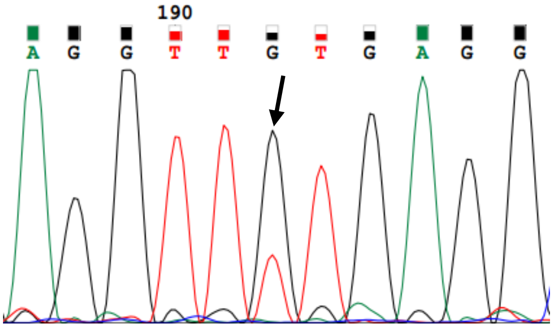

P99: TP53 c.524G>A p.R175H

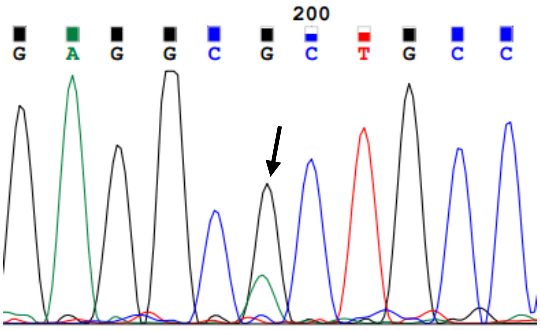

P171: TP53 c.524G>A p.R175H

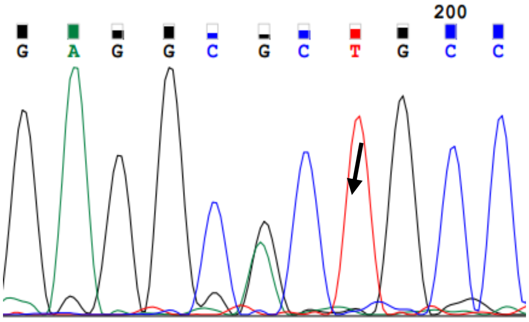

P154: TP53 c.524G>A p.R175H

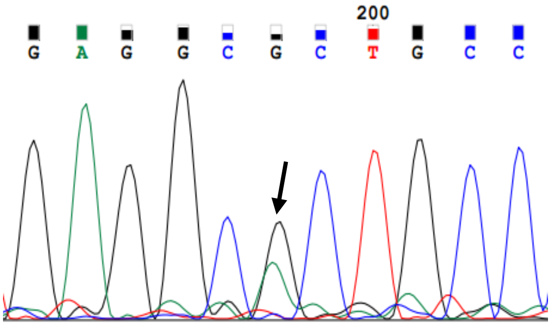

P307: TP53 c.538G>A p.E180K

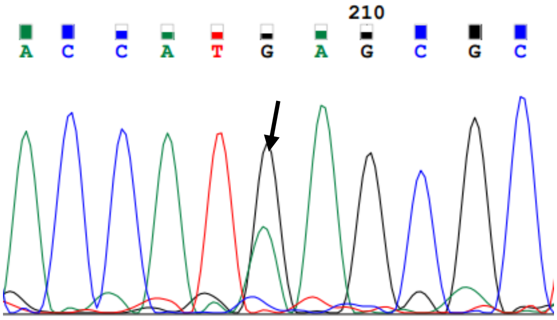

P252: TP53 c.581T>G p.L194R

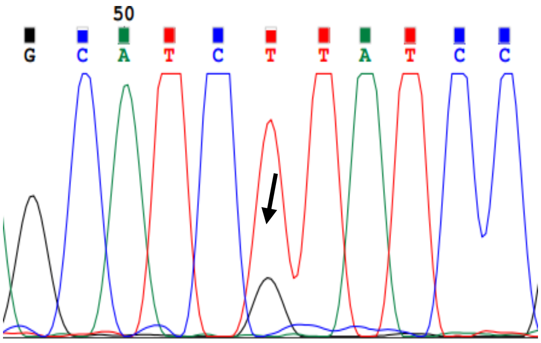

P210: TP53 c.592G>T p.E198*

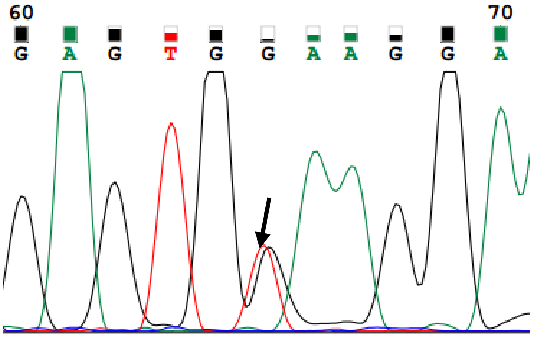

P304: TP53 c.596G>T p.G199V

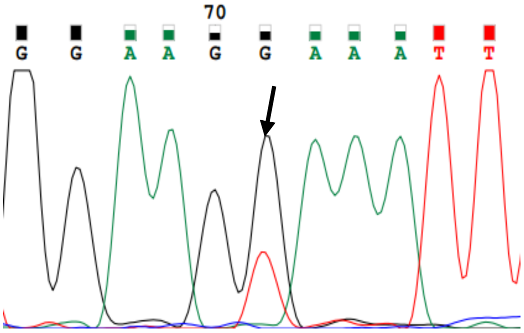

P308: TP53 c.614A>G p.Y205C

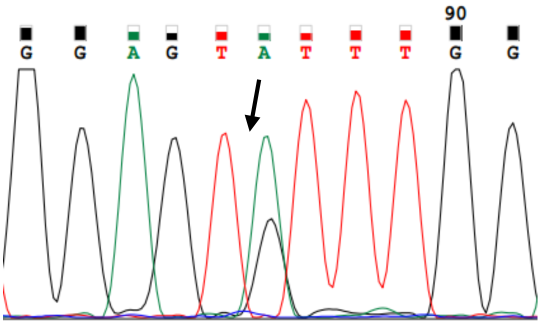

P1: TP53 c.626_627delGA p.R209KfsTer6

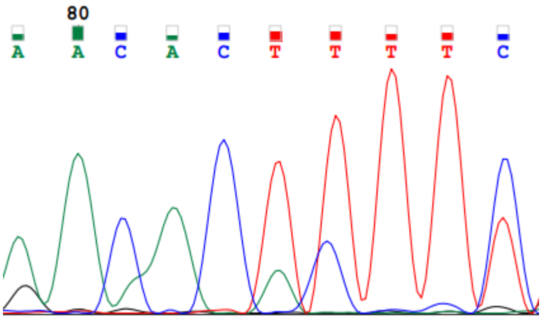

P121: TP53 c.637C>T p.R213*

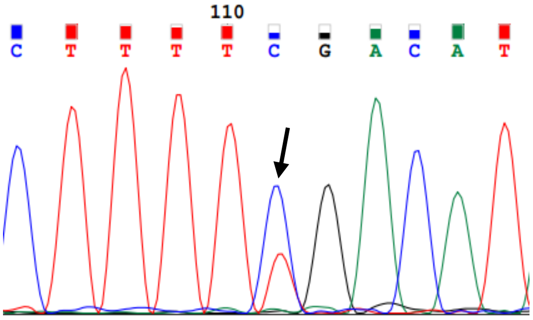

P105: TP53 c.641A>G p.H214R

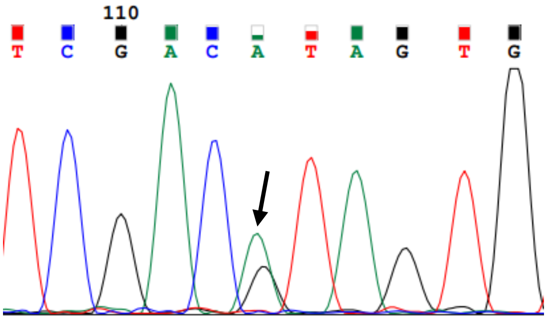

P126: TP53 c.645T>G p.S215R

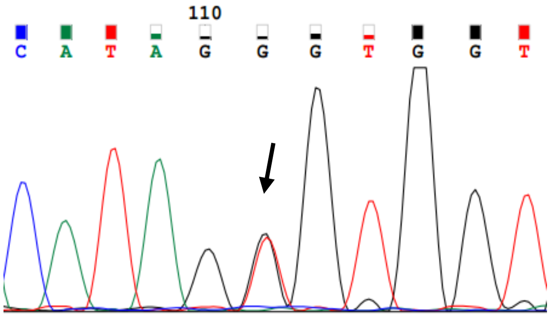

P178: TP53 c.652_654delGTG p.V218del

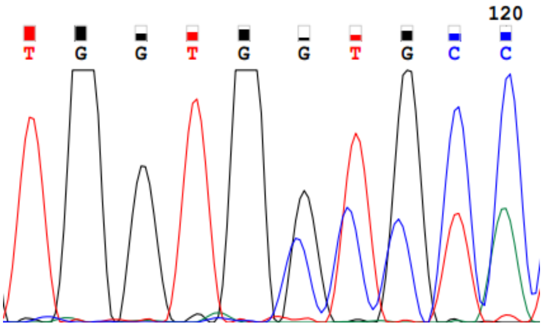

P298: TP53 c.700_702dupTAC p.Y234dup

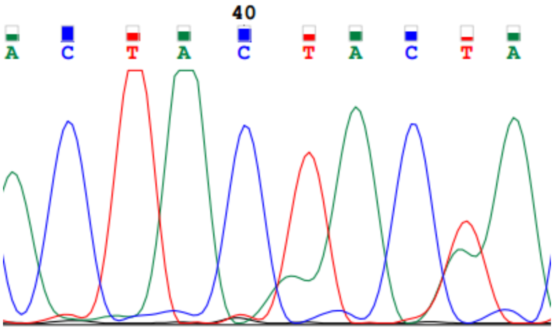

P106: TP53 c.713G>T p.C238F

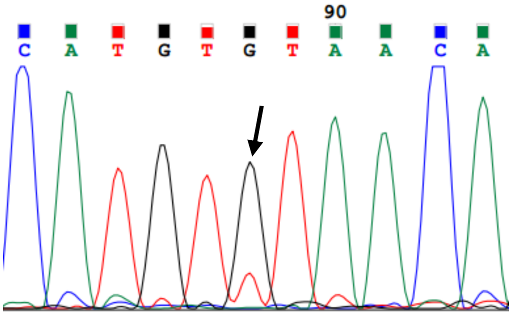

P71: TP53 c.721T>C p.S241P

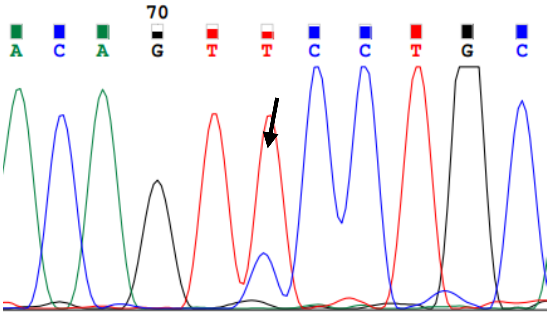

P293: TP53 c.730G>A p.G244S

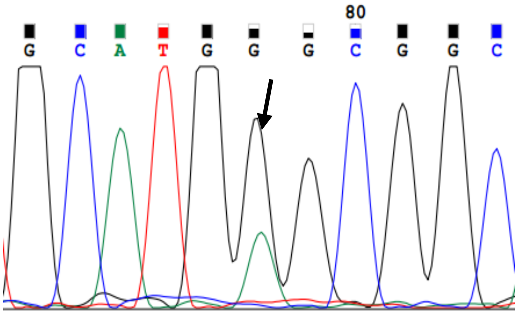

P6: TP53 c.742C>T p.R248W

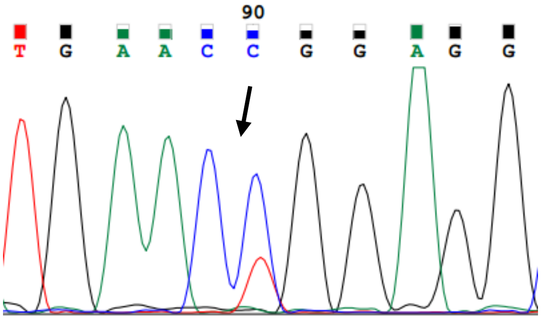

P213: TP53 c.742C>T p.R248W

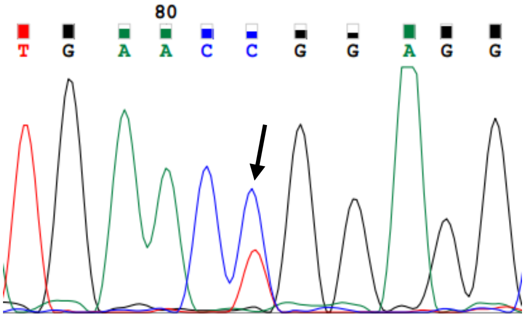

P110: TP53 c.743G>A p.R248Q

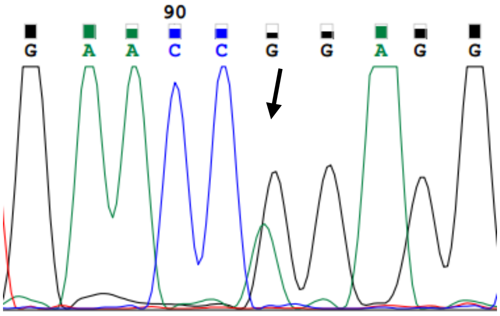

P212: TP53 c.764T>G p.I255S

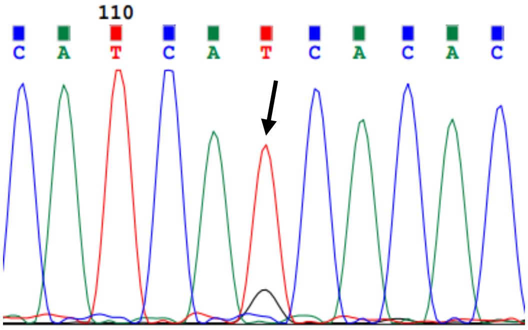

P256: TP53 c.797G>A p.G266E

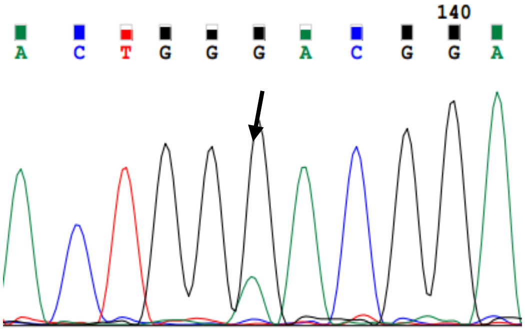

P19: TP53 c.817C>T p.R273C

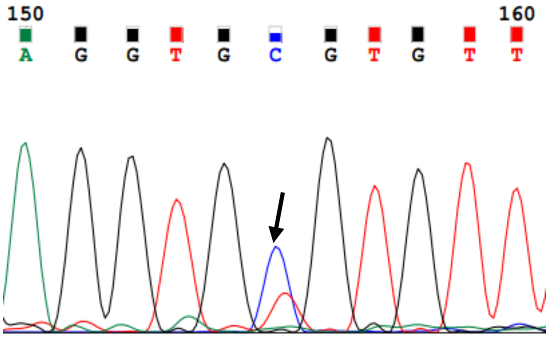

P58: TP53 c.817C>T p.R273C

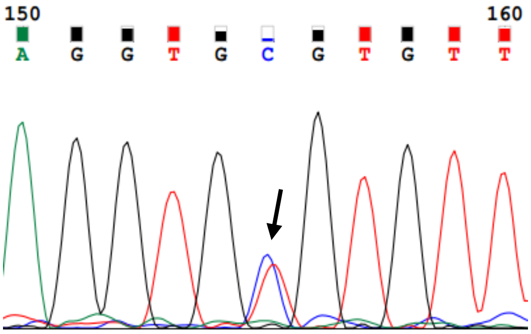

P17: TP53 c.818G>A p.R273H

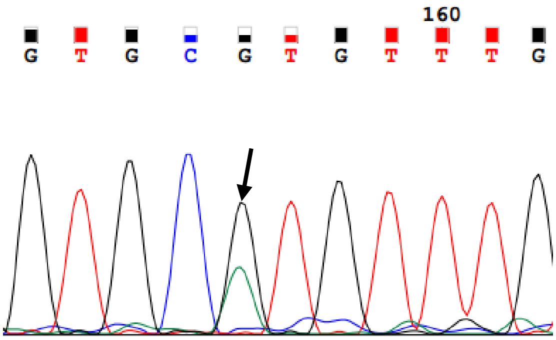

P214: TP53 c.818G>A p.R273H

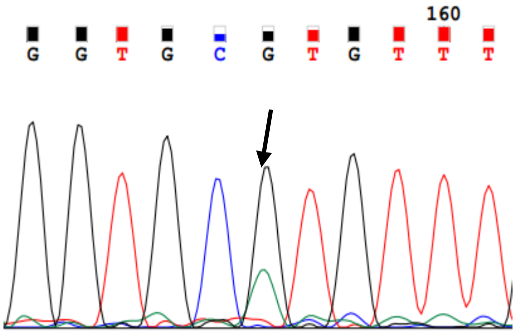

P191: TP53 c.820_821delGT p.V274LfsTer31

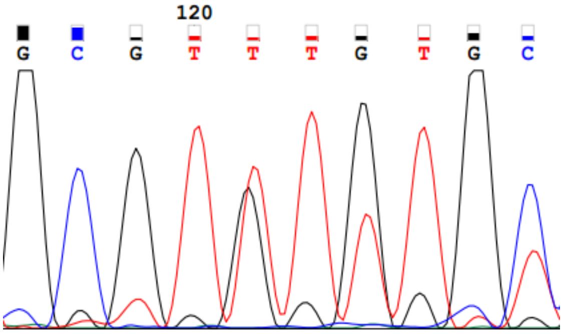

P91: TP53 c.820G>T p.V274F

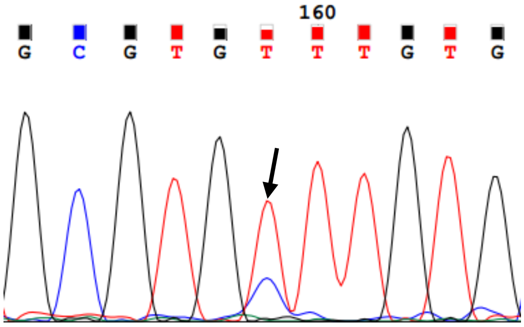

P237: TP53 c.821T>C p.V274A

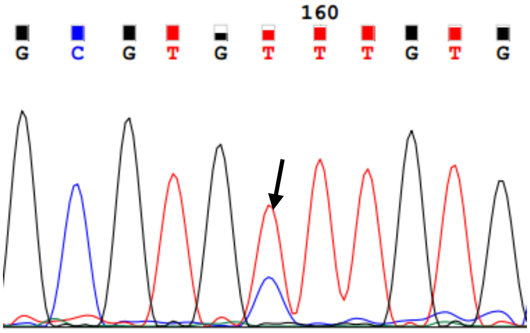

P248: TP53 c.826_831delGCCTGT p.A276_C277del

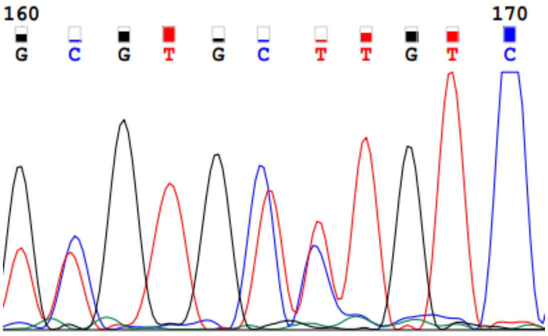

P144: TP53 c.830G>T p.C277F

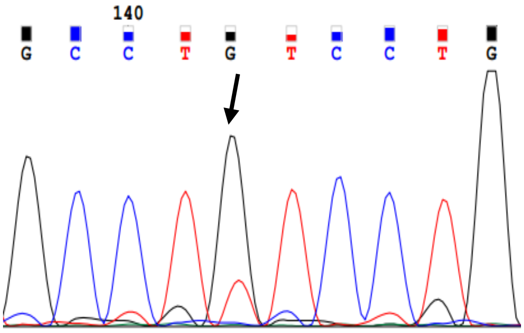

P313: TP53 c.833C>A p.P278H

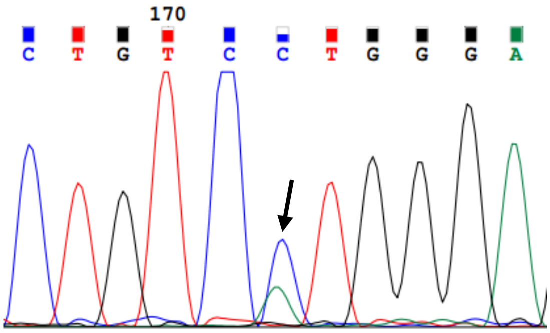

P111: TP53 c.840A>C p.R280S

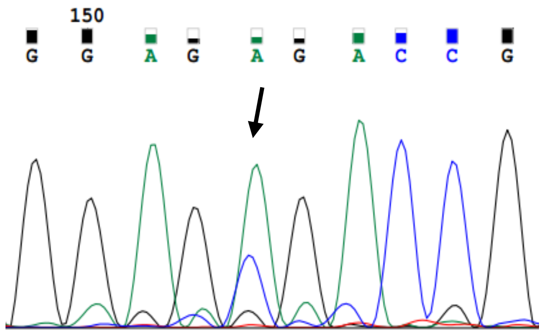

P70: TP53 c.853G>A p.E285K

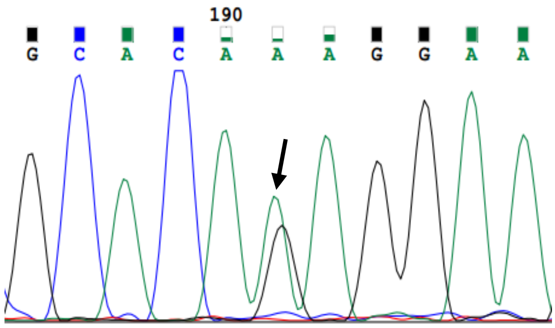

P197: TP53 c.916C>T p.R306*

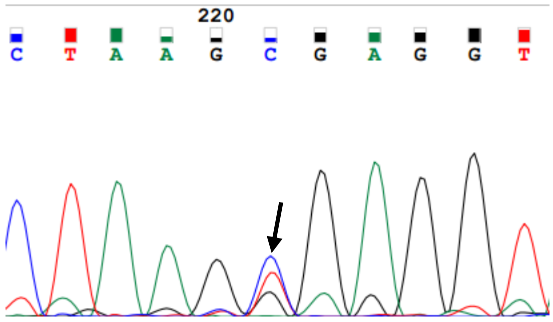

P216: TP53 c.929delA p.N310TfsTer35

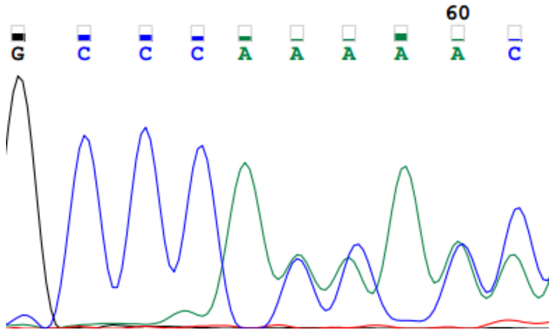

P21: TP53 c.1146delA p.K382NfsTer40

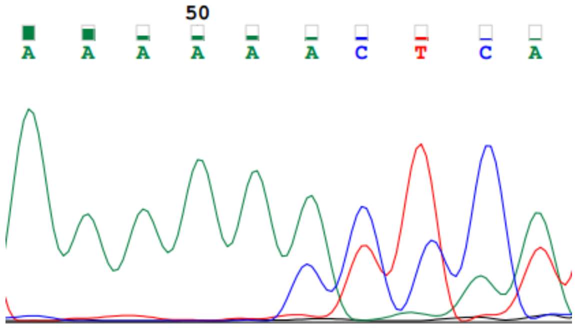

Supplement: S1 Fig — (PDF) [file pone.0203495.s001.pdf]
