# Supplementary material for: Prevalence and spectrum of AKT1, PIK3CA, PTEN and TP53 somatic mutations in Chinese breast cancer patients
Source: PLoS One. 2018 Sep 13;13(9):e0203495. doi: 10.1371/journal.pone.0203495 (PMC6136723; doi:10.1371/journal.pone.0203495)
Supplement: S2 Fig — (PDF) [file pone.0203495.s002.pdf]

SUPPLEMENTARY FIGURES

Figure S2. Verification of germline mutations in tumor/normal tissues by Sanger sequencing

P274： PIK3CA c.2198A>G p.K733R

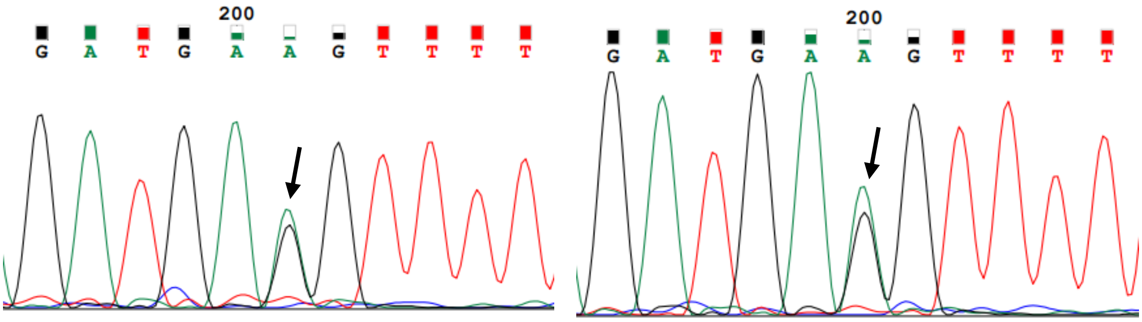

Tumor Normal

P205： PTEN c.406T>C p.C136R

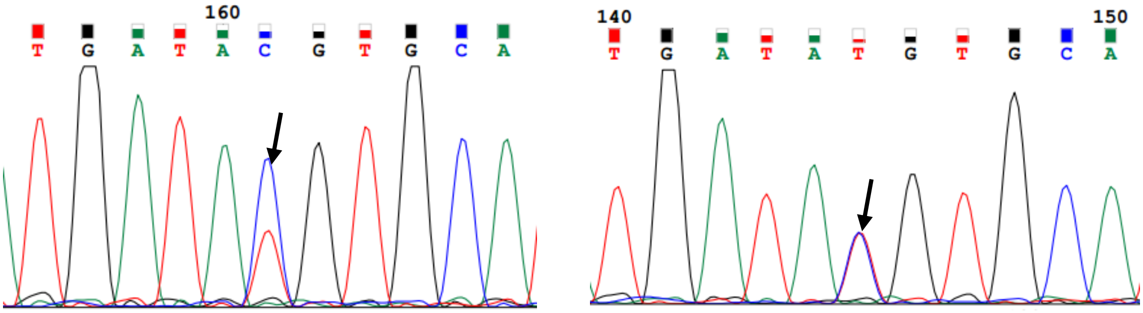

Tumor Normal

P250： PTEN c.328C>G p.Q110E

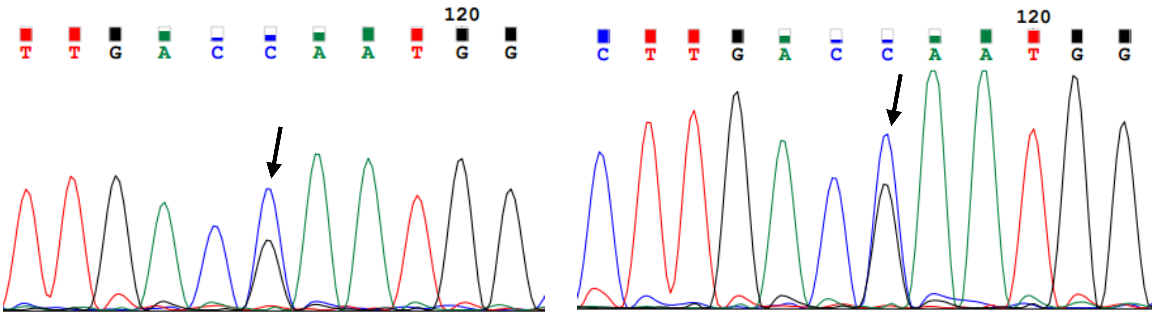

Tumor Normal

P22： TP53 c.559+1G>A

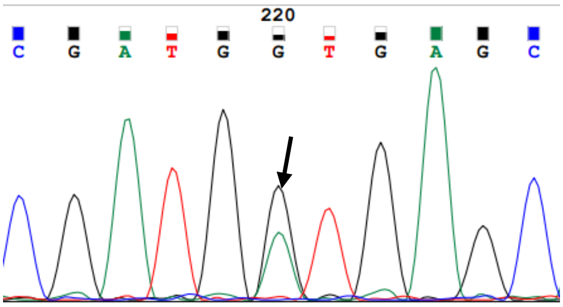

Tumor

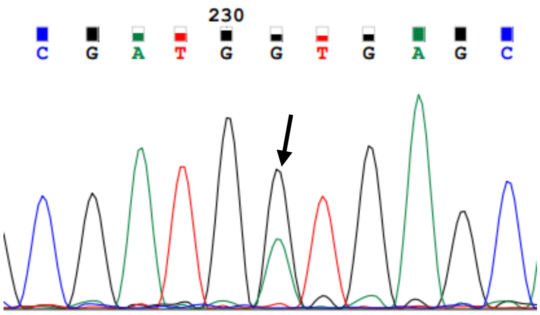

Normal

P235： TP53 c.884C>T p.P295L

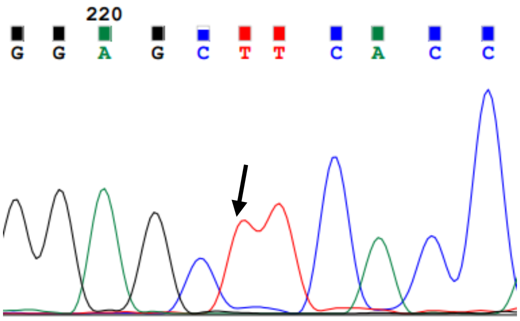

Tumor

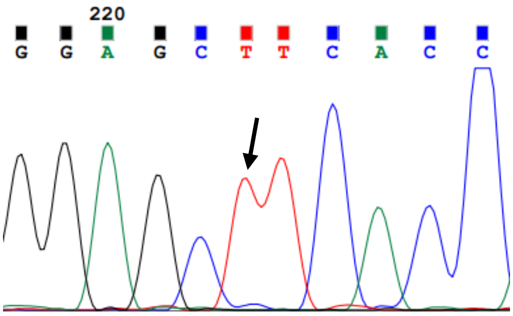

Normal

P278： TP53 c.730G>A p.G244S

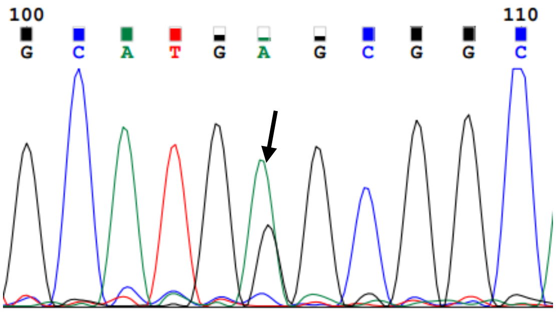

Tumor

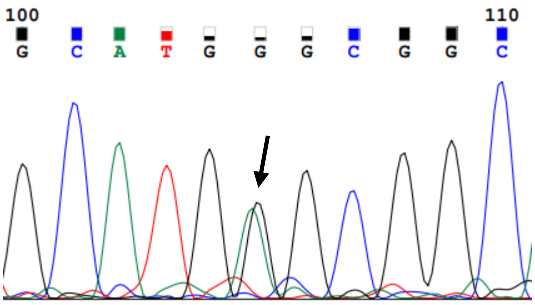

Normal
